# Immuno-modulatory nanozyme film with self-switchable activity for adaptive infection-inflammation-repair cascade in wound infection therapy and healing

**DOI:** 10.1016/j.mtbio.2026.103465

**Published:** 2026-07-15

**Authors:** Wenlong Zhang, Yilin Yuan, Jiaqi Liu, Jiang Du, Xinxin Zhao, Zhen Jin, Xianglong Zhu, Xiaojuan Huang, Dapeng Wu, Junqing Hu

**Affiliations:** a College of Medical Engineering, College of Mathematical Medicine, College of Public Health, Xinxiang Medical University, Xinxiang, 453003, China; b College of Health Science and Environmental Engineering, Shenzhen Technology University, Shenzhen, 518118, China; c Key Laboratory of Yellow River and Huai River Water Environment and Pollution Control, Ministry of Education, College of Environment, Henan Normal University, Xinxiang, 453007, China; d Department of Oral and Maxillofacial-Head & Neck Oncology, Shanghai Ninth People's Hospital, Shanghai Jiao Tong University School of Medicine, Shanghai, 200011, China; e College of Mathematics, Physics and Statistics, Shanghai University of Engineering Science, Shanghai, 201620, China

**Keywords:** Nanozyme films, Self-switchable activity, Wound infection therapy and healing, Wound dressings, Antibacterial and anti-inflammatory effect, Infection-inflammation-repair

## Abstract

The key for nanozymes to achieving sequential infection-inflammation-repair in wound infection therapy and healing lies in how to resolve the contradiction of reactive oxygen species (ROS) regulation between antibacterial therapy and anti-inflammation. Herein, a freestanding pristine CuGeO_3_ (CGO) nanozyme film (NF) composed of nanowires is first designed and simply synthesized. Such a NF with flexibility, wet tissue adhesion properties, alongside water/blood absorbing capability could serve as a wound dressing and present superb antibacterial/antibiofilm efficacy for photothermal/chemodynamic synergistic therapy of wound infection owing to its extraordinarily outstanding photothermal performance, and glutathione depletion-enhanced peroxidase-like activity to generate ROS in acidic infection microenvironments (IMEs). Attractively, it is disclosed that after completing infection therapy, the enzyme activity of this CGO NF automatically and continuously switches into catalase-like activity to eliminate ROS as IMEs switches into neutral microenvironments. This promotes the M2 phenotype polarization of macrophages to relieve inflammation and accelerate wound repair. Furthermore, the longer and entangled nanowire structure of the CGO NF prone to retention in the wound site minimizes its toxicity to healthy tissues. Overall, it is the first paradigm to leverage a photothermal NF consisting of inorganic nanomaterials only as a wound dressing. Based on such a safe, effective, activity-switchable NF dressing, adaptive infection-inflammation-repair cascade in wound infection therapy and healing has been realized, achieving 98.7% wound closure within 10 days. We believe this work will foster the therapeutic application of activity-switchable nanozymes, and provide meaningful insights into nanozymes-based wound infection therapy and healing.

## Highlights

• An activity-switchable NF to realize adaptive infection-inflammation-repair cascade in wound infection therapy and healing.
• It is the first paradigm to leverage a photothermal NF consisting of inorganic nanomaterials only as a wound dressing.
• Extraordinarily excellent photothermal performance of CGO NF from high NIR absorbance and unique porous network structure.
• Longer entangled nanowire structure of CGO NF prone to retention in the wound site minimizes toxicity to healthy tissues.
• The underlying anti-inflammatory and wound repair mechanism aroused by M2 macrophage polarization is carefully revealed.

## Graphical abstract

**Figure:**
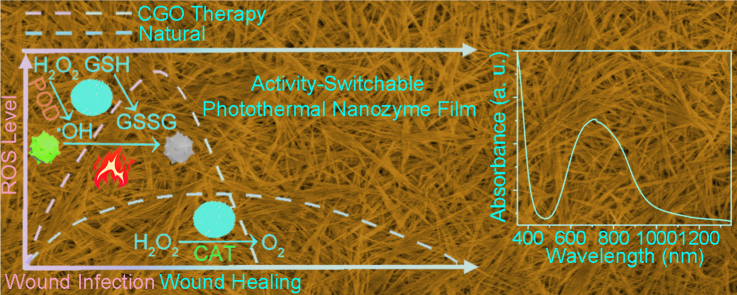

## 1 Introduction

Regulation of redox homeostasis via reactive oxygen species (ROS) generation and elimination catalyzed by nanozymes has been proposed as a promising therapeutic strategy as intensively seen in recent studies [1,2]. In this regard, nanozymes-initiated wound infection therapy or wound healing has garnered increasing interest lately, since nanozymes generating ROS are able to kill bacteria for wound infection therapy [3], or nanozymes eliminating ROS are capable of alleviating inflammation to accelerate wound healing [[4], [5], [6]]. As such, there are already a substantial amount of research concentrating on developing high-activity nanozymes for wound infection therapy and healing [[3], [4], [5], [6], [7], [8], [9], [10], [11]]. Nonetheless, their biosafety encounters challenge, given their toxicity may come from both their inherent physicochemical properties and their enzymatic activity, which will cause damage to normal tissues and cells. Besides, it is noteworthy that nanozymes alone usually cannot serve as wound dressings, restricting their practical application [12]. To overcome this hurdle, the current approach is to incorporate nanozymes into hydrogels [13], microneedles [14], or fibers [15]. Nevertheless, such an approach also demonstrates distinct limitations, including complicated synthesis routes, inconsistent distribution of nanozymes and mask of active sites leading to weakened activity, or biosafety concern from released nanozymes. Therefore, it is highly desirable to develop an easy-to-fabricate nanozyme with the ability to function as a wound dressing while maintaining excellent biosafety and high activity for wound infection therapy and healing [16].

On the other hand, an ideal wound infection therapy and healing process necessitates a sequential infection-inflammation-repair strategy involving both antibacterial therapy and anti-inflammation. To achieve this, the key lies in how to balance antibacterial therapy and anti-inflammation. The feature of wound microenvironments dynamically switches according to different therapy and healing stages. It is, thus, expected to engineer a nanozyme with switchable catalytic activity that could adapt to different microenvironments to promptly generate ROS for antibacterial therapy prior to anti-inflammation through timely eliminating ROS. This resolves the contradiction of ROS regulation between antibacterial therapy and anti-inflammation. However, most studies concerning the relationship between nanozyme activity and microenvironments focus on improving the selectivity of nanozymes, or breaking the limitation of microenvironments to eliminate or generate specific types of ROS [[17], [18], [19], [20]]. These nanozymes fail to realize both antibacterial therapy and anti-inflammation. It seems a viable route to engineer nanozymes with photothermal, magnetic hyperthermia, photodynamic or sonodynamic performance for antibacterial therapy followed by anti-inflammatory effect of nanozymes through eliminating ROS. Although with some progress [[4], [5], [6]], merely relying on these physical therapy methods, via producing localized hyperthermia (>55 ^o^C) or sufficient singlet oxygen to eradicate drug-resistant bacteria, easily induces either collateral damage to surrounding healthy tissues or incomplete bacterial eradication, owing to unclear demand of therapeutic intensity for just-right bacterial eradication. Therefore, how to achieve nanozymes-based both antibacterial therapy and anti-inflammation for safe and effective wound infection therapy and healing remains a challenge.

Most recently, intelligent regulation of ROS generation and elimination by microenvironments-activated nanozymes with self-switchable activity has emerged, as expected, as an innovative avenue to adaptive infection-inflammation-repair cascade in wound infection therapy and healing [[21], [22], [23], [24], [25]]. For instance, a microneedle bandage was developed with the dual-directional regulation of ROS generation and elimination in response to different glutathione (GSH) levels for biofilm eradication and anti-inflammatory effect [21]. A programmable hydrogel with glucose-activated self-switchable enzyme-like activity was reported for wound healing of infected diabetes [22]. However, straightforward synthesis of a nanozyme as a wound dressing with such interesting self-switchable activity has not yet been reported thus far.

**Scheme 1:**
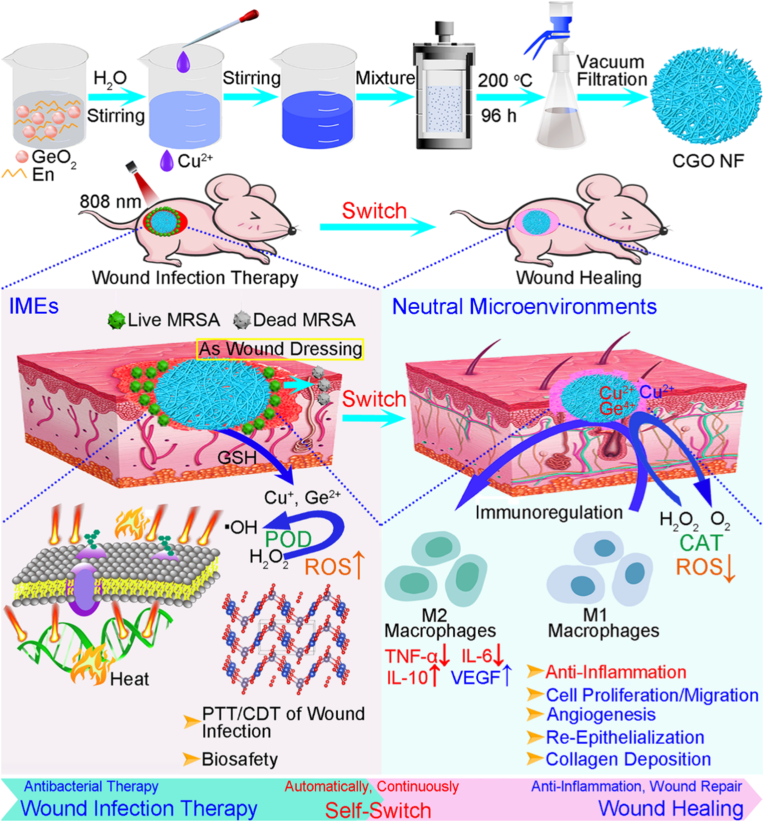
Schematic illustration of synthesized microenvironments-activated CGO NF dressing with high NIR absorbance and self-switchable activity for adaptive infection-inflammation-repair cascade in wound infection therapy and healing.

It is known that bacteria commonly yield metabolites, involving lactic acid, acetic acid and malic acid, rendering specific bacterial infection microenvironments (IMEs) acidic with pH 4.5-6.5 [26]. When the bacteria are completely eradicated, the wound would go through inflammation with such acidic IMEs switching into near-neutral microenvironments, which could act as a target for regulating ROS by pH-activated nanozymes with self-switchable activity [27]. Moreover, Cu-based nanozymes often exhibit peroxidase (POD)-like activity and exceptional photothermal performance [28]. Meanwhile, Ge-associated derivatives or nanoparticles along with Cu^2+^ have been found to own antioxidant/anti-inflammatory properties [29,30]. Nevertheless, the main bottlenecks of Cu- and Ge-based nanozymes are biosafety concerns arising from excessive Cu use and limited functionality, respectively. Additionally, assembly of 1D nanomaterials into 2D macroscopic films has been widely probed since these films generally possess high porosity, good flexibility and large specific surface area [31]. Building on the existing research and ideas, we have successfully designed and synthesized a freestanding CuGeO_3_ (CGO) nanozyme film (NF) composed of nanowires, which is derived from inorganic agents only by a controllable hydrothermal method followed by an easy vacuum filtration process. The synthesized CGO NF with flexibility, wet adhesion properties, as well as water/blood absorbing ability could act as a wound dressing that displays strong near-infrared (NIR) absorbance, enabling safe and high-efficiency photothermal antibacterial and antibiofilm therapy for wound infection. Also, in acidic IMEs, such a NF could produce ROS through its POD-like activity and deplete GSH for boosting chemodynamic therapy (CDT) of bacteria and biofilm, thereby assisting photothermal therapy (PTT) to ensure biosafety. Fascinatingly, in neutral microenvironments, its enzyme-like activity spontaneously switches into catalase (CAT)-like activity for rapid ROS elimination. This effectively promotes the polarization of macrophages from M1 to M2 phenotype, exerting anti-inflammatory effect and facilitating wound repair. This activity-switchable NF intelligently and continuously regulates the ROS level of wound microenvironments to realize both antibacterial and anti-inflammatory efficacy. Further, the CGO NF is endowed with favorable biosafety as its longer and entangled nanowires tend to be retained in the wound site. Collectively, based on such a novel microenvironments-activated NF dressing with high NIR absorbance and self-switchable activity to intelligently regulate ROS, a safe, effective and adaptive infection-inflammation-repair cascade in wound infection therapy and healing has been achieved (Scheme 1). Additionally, the intrinsic mechanism of anti-inflammatory and wound repair activity aroused by M2 macrophage polarization was carefully revealed.

## 2 Results and discussion

### 2.1 Synthesis and characterization of CGO NF

**Fig. 1:**
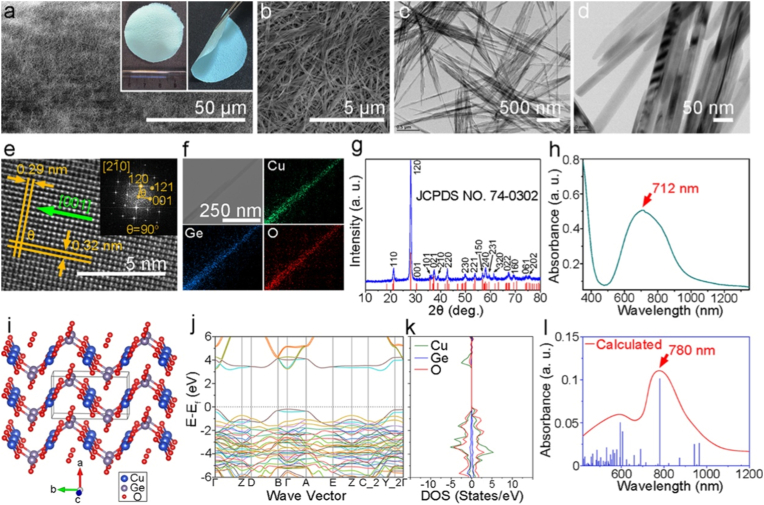
Characterization, optical absorption of the CGO NF and theoretical calculations. (a,b) SEM and (c,d) TEM images of the CGO NF in different magnifications. Insets in (a): photos of a freestanding and flexible CGO NF. (e) HRTEM and (f) STEM-EDS elemental mapping images of an individual CGO nanowire. Inset in (e) is the corresponding FFT pattern. (g) XRD pattern and (h) UV-Vis-NIR diffuse reflectance spectrum of the CGO NF. (i) Crystal structure, (j) energy band structure, (k) DOS and (l) calculated absorption spectrum of CGO.

The optimized CGO NF was synthesized by the hydrothermal method with the assistance of ethylenediamine (En) at 200 ^o^C for 96 h and subsequent further vacuum filtration (Scheme 1; Figs. S1–S4; see Materials and Methods section in the Supporting Information). Such a film could be peeled off from the filter membrane to yield freestanding structure. As exhibited in the insets of Fig. 1a as well as Figs. S5 and S6, a blue round lightweight paper-like freestanding film with a diameter of around 40 mm and thickness of about 0.2 mm was obtained and could be easily bent without any damage, demonstrating sufficient flexibility. The scanning electron microscopy (SEM) images illustrate that the obtained film is composed of numerous disordered nanowires that entangle together to generate a porous network structure (Fig. 1a and b). Consistent with the SEM observation, nitrogen adsorption-desorption isotherms of this film reveal the characteristic hysteresis loop coinciding with the mesoporous structure, and pore distribution analysis identifies the mean pore size is about 14.6 nm (Fig. S7). It is such a unique porous network structure that endows this film with flexibility, large numbers of active sites, superior adsorption ability and simultaneously high heat transport capability [32,33]. The transmission electron microscopy (TEM) images further reveal that these well-defined and uniform nanowires are straight and smooth with a diameter of 22.3 ± 5.9 nm and length up to 2.3 ± 0.7 μm (Fig. 1c and d; Figs. S3 and S4). From high-resolution transmission electron microscopy (HRTEM) images, lattice spacings of 0.29 and 0.32 nm are observed on an individual nanowire, corresponding to the (001) and (120) planes in orthorhombic CGO, respectively, and the nanowire grows along the [001] direction (Fig. 1e; Fig. S8). The inset in Fig. 1e is the corresponding fast Fourier transformation (FFT) pattern, verifying the single-crystalline nature. Similarly, the selected area electron diffraction (SAED) pattern taken from an individual CGO nanowire is shown in Fig. S9, which could be assigned to that of the [1 $\bar{1}$ 0] zone axis of the orthorhombic CGO crystal. Moreover, the scanning transmission electron microscopy (STEM)-energy disperse spectroscopy (EDS) elemental mapping images prove the homogeneous distribution of Cu, Ge and O elements along the nanowire (Fig. 1f). Further EDS spectrum collected from a spot on an individual CGO nanowire reveals an estimated atomic ratio of 1.03:1 for Cu and Ge (Fig. S10 and S11). Also, quantitative analysis of the EDS spectrum recorded from a large region of the CGO NF demonstrates that the Cu/Ge atomic ratio is estimated to be 0.99:1 (Fig. S12 and S13). Furthermore, X-ray diffraction (XRD) analysis on this nanowire film displays all peaks assigned to the orthorhombic CGO (JCPDS 74-0302), and a strongly preferred orientation along (120) plane, in agreement with the result of HRTEM (Fig. 1g). These findings confirm successful synthesis of the CGO NF composed of nanowires. Notice that as for synthesizing the CGO NF, the hydrothermal method is controllable (Figs. S1–S4). Especially, the subsequent vacuum filtration process is easy and readily amendable to scaling up through choosing large-size filter membrane and elevating the concentration and volume of nanowire dispersions for filtration [32], favoring practical clinical application of this film.

### 2.2 Optical absorption of CGO NF and theoretical calculations

The optical absorption properties of this synthesized CGO NF were further studied. Interestingly, the CGO NF displays a high absorption peak, comparable to the localized surface plasmon resonance peak of Au nanorods [34], at approximately 712 nm (∼1.7 eV) in the NIR region from the UV-Vis-NIR diffuse reflectance spectrum (Fig. 1h). Such a high absorption peak enables excellent photothermal performance under 808 nm laser irradiation.

Fig. 1i and S14 illustrate CGO has an orthorhombic unit cell crystal structure, which is constructed by edge-sharing CuO_4_ squares and corner-sharing GeO_4_ tetrahedra stacked along c direction. To further evidence above optical absorption properties of the CGO NF, density functional theory calculations on CGO were performed. Seen from the typical energy band structure (Fig. 1j), the valence band and conduction band are well separated with a bandgap of 3.3 eV, demonstrating CGO is semiconducting. The corresponding density of state (DOS) shown in Fig. 1k unveils the top of valence band is comprised of O and Cu, while the bottom of conduction band consists of Cu, agreeing well with previous theoretical study [35]. As expected, the calculated optical absorption spectrum (Fig. 1l) shows that there is a significant absorption peak located at about 780 nm (∼1.6 eV) in the NIR region, approximately in accordance with our experimental measurement and previous theoretical calculations [[35], [36], [37]]. According to previous reports, the feature peak centered at 1.4-2.3 eV (∼539-886) nm in the absorption spectrum of CGO is derived from the d-d electron transition [[35], [36], [37]].

### 2.3 Photothermal performance of CGO NF

**Fig. 2:**
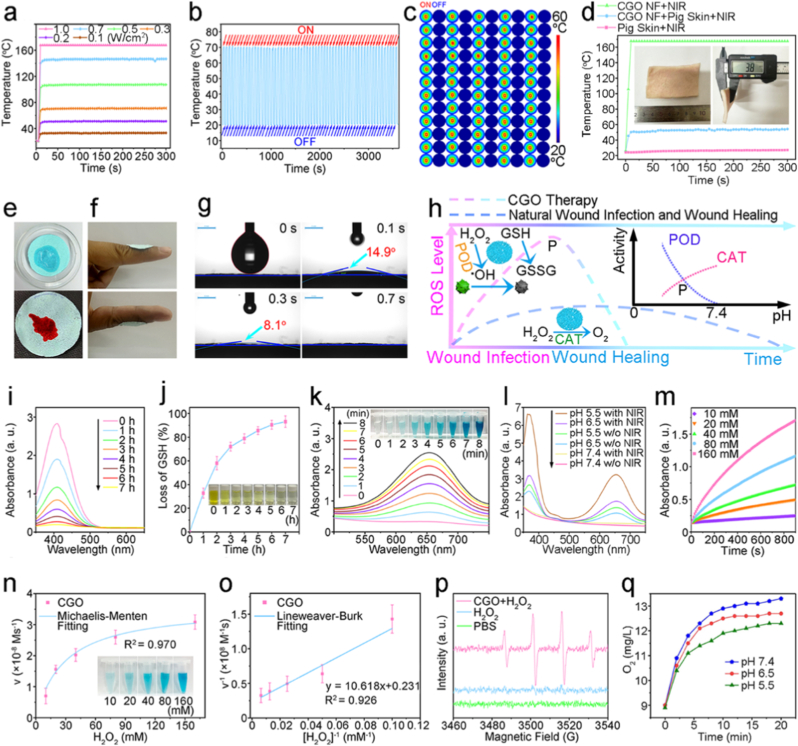
Properties of the CGO NF. (a) Temperature elevation curves of the CGO NF under 808 nm NIR laser irradiation at various power densities. (b) Photothermal stability of the CGO NF under 808 nm laser irradiation of 0.3 W/cm^2^ through sixty on/off cycles (one cycle is that the CGO NF was irradiated for 30 s and then the NIR laser was turned off for 30 s to cool the NF to room temperature) and (c) corresponding IR thermal imaging. (d) Temperature elevation curves of a CGO NF covered with a pig skin, the CGO NF, and the pig skin under 808 nm laser irradiation of 1.0 W/cm^2^. Insets showing photos of the pig skin and its thickness. Photos exhibiting (e) the H_2_O and blood absorbing capability of the CGO NF and (f) the adhesion properties of the CGO NF to wet human skin. (g) Contact angle of water on the CGO NF. (h) Schematic illustration of the pH-activated CGO NF with self-switchable activity. (i) UV-Vis absorption spectra of GSH incubated with DTNB in PBS (pH 7.4) treated with the CGO NF and (j) corresponding GSH loss ratio and color changes. (k) Time-dependent absorption changes of TMB and corresponding color changes in the presence of the CGO NF and H_2_O_2_ in PBS (pH 5.5). (l) UV-Vis absorption spectra of TMB in the presence of the CGO NF and H_2_O_2_ in PBS under different pH with/without 808 nm NIR laser irradiation. (m) Time-dependent absorbance changes of TMB at 652 nm in the presence of the CGO NF with various H_2_O_2_ concentrations in PBS (pH 5.5). (n) Michaelis-Menten kinetic analysis (insets: photos of solutions with varying H_2_O_2_ concentrations) and (o) Lineweaver-Burk plotting of the CGO NF with H_2_O_2_ as a substrate. (p) ESR spectra indicating the •OH generation by the CGO NF at pH 5.5. (q) O_2_ generation from solutions in the presence of the CGO NF and H_2_O_2_ in PBS with varied pH. (For interpretation of the references to color in this figure legend, the reader is referred to the Web version of this article.)

Motivated by such a strong absorption peak in the NIR region, the photothermal performance of the CGO NF was explored. After 808 nm NIR laser irradiation at power densities of 0.1, 0.2, 0.3, 0.5, 0.7, and 1 W/cm^2^ for 5 min, the temperature of the CGO NF increased by 12.4, 30.2, 50.5, 86.5, 125.5 and 146.3 ^o^C, respectively, clearly indicating the photothermal effect is dependent on the power density (Fig. 2a). Note that a very rapid temperature soar of the CGO NF occurs merely within 5 s once under irradiation and temperature swiftly reaches the plateau. This exceptional photothermal effect may be ascribed to the unique porous network structure of the CGO film, as stated above, thus ensuring high heat transport during the photothermal conversion process [13]. Even after sixty on-off cycles of NIR irradiation, the maximum temperature of the CGO NF consistently remains around 70 °C as verified by the infrared thermal imaging, revealing its outstanding photothermal stability (Fig. 2b and c). In particular, the temperature of the CGO NF presents fast rise and fast fall, rendering the CGO NF ideal for precise and safe PTT of wound infection, avoiding thermal damage to surrounding normal tissues.

Next, the photothermal performance of the CGO NF in simulated environments of hyperglycemia, GSH, and acidity was assessed. As presented in Figs. S15–S17, the CGO NF exhibits a significant temperature rise in such environments. Further, the deep tissue photothermal performance of the CGO NF was assessed using a pig skin (the insets in Fig. 2d). A CGO NF covered with the pig skin was exposed to 808 nm laser irradiation of 1.0 W/cm^2^. Seen from Fig. 2d and S18, efficient heating of the CGO NF is achieved without causing significant temperature rise inside the pig skin, even though the thickness of the pig skin reaches 3.8 mm, suggesting safe and effective NIR penetration through deep tissue. These results demonstrate that the CGO NF is a promising candidate for PTT of deep diabetic wounds.

### 2.4 Water/blood absorbing capability and wet tissue adhesion properties of CGO NF

In general, a suitable wound dressing necessitates capability of absorbing exudate fluid and blood, and should present tissue adhesion to seal the wound as well. The CGO NF could rapidly absorb water and blood, as displayed in Fig. 2e and Fig. S19. Also, as anticipated, the CGO NF, shows moderate adhesion to the wet finger surface without detachment, even after shaking (Fig. 2f). Notably, this moderate wet adhesion properties of the CGO NF enable its effortless removal from wound to prevent secondary damage.

To explore why water/blood absorption and wet tissue adhesion occur, the water-wetting behavior on such a NF was investigated. When a tiny water droplet contacts the surface, it instantly spreads out and infiltrates into the NF with the contact angle quickly decreasing to nearly 0^o^ within 0.7 s (Fig. 2g), unveiling the superhydrophilicity of the CGO NF. This leads to rapid water/blood absorption along with further wet adhesion, making the CGO NF compatible for use as a wound dressing.

Moreover, it is observed that the CGO NF consisting solely of inorganic nanomaterials is easily partially dispersed in the solution without ultrasonic treatment (Fig. S20), and could be easily dispersed in physiological solutions such as phosphate buffered saline (PBS) and Dulbecco's Modified Eagle's Medium via sonication (Fig. S21). Photos for the supernatants of the CGO NF ultrasonically dispersed in water with typical Tyndall effect indicate its certain hydrophilicity and dispersity (Fig. S22). Additionally, when gradually increased amounts of water or PBS were dropped onto its surface, the CGO NF became more and more muddy and slurry-like (Fig. S23 and S24). All these demonstrate that our work actually introduces a special material system that draws inspiration from both the nanoscale materials and the macroscopic dressing. That is to say, the CGO NF could not only function as a wound dressing, but also serve like a nanoagent. For this reason, in this study, the following catalytic activity (POD, CAT, and GSH depletion), intracellular O_2_ generation, HIF western blot, flow cytometry of macrophage polarization, RNA sequencing, qRT-PCR, anti-inflammatory western blot, and cell proliferation/migration assays were performed using ultrasonically dispersed samples to ensure sample activity and efficacy to be quantitatively analyzed and these chosen concentrations were set to better reveal the comprehensive performance of CGO NFs. In contrast, in vitro antibacterial/antibiofilm and ROS tests, as well as in vivo wound therapy experiments were conducted using CGO NFs as freestanding films. This film testing format was primarily for qualitative validation of CGO NF therapeutic efficacy, and more importantly, to be as close to practical application scenarios as possible (Table S1). Notably, data from different assays cannot be directly compared, and all subsequent cross-functional quantitative analyses must adopt a unified normalization criterion.

### 2.5 pH-activated CGO NF with self-switchable activity

As highlighted above, constructing a microenvironments-activated activity-switchable nanozyme to intelligently regulate ROS generation and elimination emerges as a promising avenue for adaptive infection-inflammation-repair cascade in wound infection therapy and healing. It is expected to elevate ROS level during the wound infection stage in the acidic microenvironments for antibacterial therapy, and at the meantime, to reduce ROS level over the wound healing stage under the neutral microenvironments to relieve inflammation. Given the dynamic pH change of wound microenvironments across wound infection therapy and healing, as well as the characteristic of nanozymes with multienzyme activity [18,20], we hope the CGO NF possesses POD-like activity under acidic conditions and GSH depletion capacity, thereby augmenting ROS production for GSH depletion-enhanced CDT against wound infection. Meanwhile, it should switch to CAT-like activity under neutral conditions to reduce ROS level for inflammation relief (Fig. 2h).

To explore the ability of the CGO NF to intelligently regulate ROS, we initially evaluate its GSH depletion capacity utilizing 5,5′-dithiobis (2-nitrobenzoic acid) (DTNB) as a probe (Fig. 2i and j). Upon the addition of the CGO NF (ultrasonic dispersion), the intensity of characteristic peaks arising from the combination of GSH and DTNB progressively diminishes, and these peaks slowly disappear over time, accompanied by the transformation from the bright yellow GSH to colorless glutathione disulfide (see the insets in Fig. 2j), demonstrating the GSH depletion capability of the CGO NF. Afterward, typical colorimetric reactions using hydrogen peroxide (H_2_O_2_) as a substrate and 3,3′,5,5′-tetramethylbenzidine (TMB) as an indicator were performed to appraise the POD-like activity of this CGO NF in acidic environments, as nanozymes with POD-like activity could oxidize TMB to blue-colored oxidized TMB (oxTMB). As depicted in Fig. 2k, the characteristic absorption peaks of oxTMB at 652 nm increase significantly with time after the addition of the CGO NF (ultrasonic dispersion) to the mixed solution of H_2_O_2_ and TMB at pH 5.5. Meantime, the color of the solution gradually becomes darker (the insets of Fig. 2k). This manifests the superior POD-like activity of the CGO NF in acidic environments. Considering the pH-activated activity switching and photothermal response of this CGO NF, the effects of pH and localized heat on its catalytic activity were also evaluated. As seen from Fig. 2l, in the presence of H_2_O_2_ and the CGO NF (ultrasonic dispersion) without NIR laser irradiation, pronounced characteristic absorption peaks at pH 5.5 and 6.5 appear, and the peak intensity at pH 5.5 is higher than that at pH 6.5. No obvious absorbance is observed at pH 7.4. This result illustrates the CGO NF has no obvious POD-like activity around neutral pH, and its POD-like activity enhances as pH decreases (Fig. 2h, inset), similar to many reported POD-like nanozymes [18,20]. Furthermore, under the 808 nm laser irradiation, distinctly boosted peak intensity could be observed at pH 5.5 and 6.5, suggesting NIR-induced heat could efficiently improve the POD-like activity of the CGO NF. To further assess the POD-like activity of the CGO NF quantitatively, the steady-state kinetics assay was studied with H_2_O_2_ as the reaction substrate at pH 5.5 (Fig. 2m–o). The steady-state kinetics follows typical Michaelis-Menten kinetics equation, and the Michaelis-Menton constant (*K*_*m*_) and maximum reaction velocity (*V*_*max*_) of the CGO NF are calculated to be 45.97 mM and 4.33 × 10^-8^ M s^-1^, respectively. Moreover, the electron spin resonance (ESR) spectra shown in Fig. 2p present a remarkable hydroxyl radical (•OH) signal for the CGO NF (ultrasonic dispersion) in the presence of H_2_O_2_ in acidic environments, further confirming the POD-like activity of the CGO NF. On the other hand, to test the CAT-like activity of the CGO NF in decomposing H_2_O_2_ into oxygen (O_2_), O_2_ generation was subsequently monitored through adding the CGO NF (ultrasonic dispersion) to H_2_O_2_ solutions at different pH. Distinct and stable O_2_ evolution, which shows gradual enhancement with the increasing pH from 5.5 to 7.4, could be observed (Fig. 2q), corroborating the CAT-like activity of the CGO NF in decomposing H_2_O_2_ to generate O_2_ improves with increasing pH, reaching optimal activity at neutral pH (Fig. 2h, inset). This neutral preferred activity is in accordance with most of reported CAT-like nanozymes [18]. From above results, the CGO NF is verified to simultaneously own both the POD-like activity and CAT-like activity at acidic pH, with the POD-like activity peaking under acidic conditions but vanishing under neutral environments, whereas the CAT-like activity minimizes in acidic settings but maximizes in neutral environments (Fig. 2h, inset). Collectively, such seemingly disharmonious multienzyme activity of the CGO NF enables pH-switchable catalytic activity under acidic or neutral conditions, facilitating intelligent modulation of the dominant catalytic behavior switched between ROS production and depletion in response to pH variations of microenvironments (Fig. 2h). ROS production is able to kill bacteria for wound infection therapy, while ROS depletion is capable of alleviating inflammation to accelerate wound healing, thus enabling adaptive infection-inflammation-repair cascade in wound infection therapy and healing. Similar pH-dependent switches from POD activity to CAT activity could be observed in other nanozymes [22,24,27,[38], [39], [40]].

To elucidate the origins of this pH-switchable catalytic activity and GSH depletion capability of the CGO NF, the POD-like activity and CAT-like activity, as well as GSH depletion capability of bulk GeO_2_ were also researched. As shown in Figs. S25–S28, bulk GeO_2_ has pronounced GSH depletion capability, but is hardly able to exert POD-like activity and CAT-like activity. Previous studies have demonstrated nanostructured GeO_2_ exhibits pH-dependent POD-like activity deprived from forming a ternary intermediate complex with H_2_O_2_ and TMB rather than the generation of •OH [[41], [42], [43]]. Considering previous studies and our investigations together, a possible reaction mechanism is described by the following Equations (1)-(7) [30,41,[44], [45], [46], [47]]:

(1) $${\text{Cu}}^{2+}+\text{GSH}\;\to \;{\text{Cu}}^{+}+\text{GSSG}+H^{+}$$

(2) $${\text{Ge}}^{4+}+\text{GSH}\;\to \;{\text{Ge}}^{2+}+\text{GSSG}+H^{+}$$

(3) $${\text{Cu}}^{+}+H_{2}O_{2}+H^{+}\;\to \;{\text{Cu}}^{2+}+\cdot \text{OH}+H_{2}O$$

(4) $${\text{Cu}}^{2+}+H_{2}O_{2}\;\to \;{\text{Cu}}^{+}+HO_{2}^{\cdot }+H^{+}$$

(5) $$HO_{2}^{\cdot }\;\to \;H_{2}O_{2}+O_{2}$$

(6) $$HO_{2}^{\cdot }+\cdot \text{OH}\;\to \;H_{2}O+O_{2}$$

(7) $$\text{TMB}+H_{2}O_{2}\overset{\text{CGO}}{\to }\;\text{oxTMB}+H_{2}O$$

Specifically, as displayed in Equations (1) and (2), GSH in IMEs could react with the CGO NF and reduce Cu^2+^ and Ge^4+^ to Cu^+^ and Ge^2+^, respectively, resulting in GSH depletion. It is known that oxidation potential of •OH decreases as the pH increases [48]. Thus, the acidic pH favors the occurrence of Fenton-like reaction by Cu^+^, conductive to the acidity-dependent POD-like activity (Equation (3)). Conversely, the elevated pH is beneficial for electron transfer from H_2_O_2_ to Cu^2+^, which is responsible for the CAT-like activity (Equations (4)-(6)). Note that the POD-like activity and CAT-like activity mediated by the CGO NF concurrently present at acidic pH as Equations (3)-(6) coexist. The above competition between POD-like activity and CAT-like activity could be explained by the amount of produced H $O_{2}^{\cdot }$. In detail, at lower acidic pH, the generation rate of •OH is faster than that of O_2_, contributing to dominant POD-like activity of the CGO NF. With increasing pH, the overproduced H $O_{2}^{\cdot }$ is capable of not only facilitating the O_2_ generation (Equation (5)), but also consuming •OH (Equation (6)), presenting increasingly decreased POD-like activity and enhanced CAT-like activity. In addition, the CGO NF, like nanostructured GeO_2_, is likely to form a ternary intermediate complex with TMB and H_2_O_2_ to exhibit POD-like activity without generation of •OH (Equation (7)). Crucially, in simulated diabetic wound environments, the CGO NF shows robust pH-dependent POD-like activity, CAT-like activity, along with GSH depletion ability as well, suggesting potential application of its self-switchable enzyme activity in diabetic wound therapy (Figs. S29–S31).

### 2.6 Biosafety of CGO NF

**Fig. 3:**
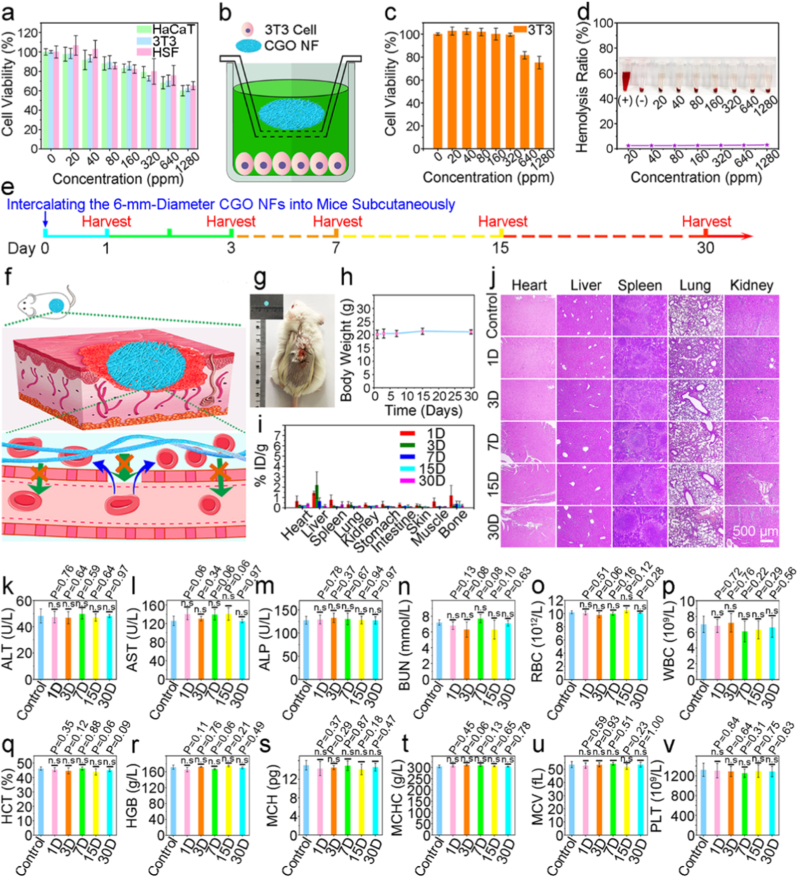
Biosafety of the CGO NF. (a) Relative cell viability of HaCaT, 3T3 and HSF cells after incubation with different concentrations of the CGO NF. (b) Schematic illustration of the transwell insert system: the CGO NF and 3T3 cells are in the upper and lower chambers, respectively. (c) Relative cell viability of 3T3 cells after incubation with varying concentrations of the CGO NF in the transwell insert. (d) The photos and percentage of hemolysis with different concentrations of the CGO NF. Schematic illustration of (e) biosafety evaluation and (f) biosafety in vivo. (g) Photos of the intercalated CGO NF (inset) and mouse biosafety evaluation model. (h) Time-dependent body weight curve within a period of 30 days after intercalation of the CGO NFs. (i) Biodistribution of Cu in major organs at different time points post intercalation of the CGO NFs. (j) H&E-stained histological images of major organs at different time points post intercalation of the CGO NFs. (k-n) Blood biochemistry and (o-v) complete blood counts of mice harvested at different time points post intercalation of the CGO NFs. n. s indicates not significant difference versus Control group.

As stressed above, a high degree of biosafety is a prerequisite for the application of nanozymes in wound infection therapy and healing. To certify the in vitro biosafety of the CGO NF, its cytotoxicity was first assessed via the cell counting kit-8 (CCK-8) assay, and different concentrations of the CGO NF by ultrasonic dispersion were co-cultured with human keratinocyte cell line HaCaT, 3T3 and human skin fibroblast (HSF) cells, respectively (Fig. 3a). The results of the CCK-8 assay demonstrate that HaCaT, 3T3 and HSF cells maintain high cell viability when the concentration of the CGO NF is lower than 640 ppm. To further simulate the real interaction between the CGO NF and tissues, the cytotoxicity of the CGO NF on 3T3 cells was examined in a transwell insert system without ultrasonic dispersion (Fig. 3b). As clearly seen from Fig. 3c, the viability of 3T3 cells is beyond 75% even when the concentration is up to 1280 ppm, implying the absence of cytotoxicity for the CGO NF in a broader range of concentrations. Moreover, the hemolysis assay was performed to assess the impact of the CGO NF on red blood cells (Fig. 3d). Compared to the negative and positive controls, the hemolysis ratios in all the concentration groups (ultrasonic dispersion) are less than 4%, indicating favorable hemocompatibility. These results collectively verify that the CGO NF possesses satisfactory biocompatibility for application in wound infection therapy and healing.

As for the in vivo biosafety of the CGO NF, previous studies have shown that the emergence of degradable and clearable inorganic nanomaterials offers the opportunity to prevent their long-term toxicity [49]. Conversely, in this work, we have engineered and constructed a less degradable NF as a wound dressing composed of longer and entangled nanowires that hardly enters bloodstream from the wound for circulation (Fig. S20; S32-S35), so as to impair its toxicity for normal tissues to the utmost [50,51], as schematically illustrated in Fig. 3f. To appraise whether the CGO NF yields toxicity and side effect in vivo, CGO NFs were subcutaneously intercalated into healthy mice (one NF was intercalated into each mouse), and these mice were euthanized to harvest major organs and blood at the 1st, 3rd, 7th, 15th and 30th day (Fig. 3e–g). Note that after intercalation, the body weight of mice has no significant change (Fig. 3h). Next, the biodistribution of CGO was investigated through testing the Cu and Ge contents in main organs at varying time points post intercalation. As expected, Cu and Ge contents in all tested organs is very low throughout the evaluation period, and increasingly reduces with time (Fig. 3i; Fig. S36). This extremely restricted biodistribution and the subsequent body clearance maximally decrease the exposure of CGO to healthy tissues, hence guaranteeing the in vivo biosafety of the CGO NF. Moreover, hematoxylin and eosin (H&E) staining of major organs at all time points post intercalation of the CGO NFs does not display any trace of inflammation or damage (Fig. 3j). Beyond that, compared with untreated mice, all hematological biomarkers remain normal after intercalation (Fig. 3k–v). Accordingly, both in vitro and in vivo measurements evidence the desirable biosafety of the CGO NF for potential application in wound infection therapy and healing. It should be noted that only the short-term biosafety of the CGO NF was investigated in this study. Long-term cumulative release of Cu/Ge ions may lead to their excessive accumulation in tissues and subsequent chronic inflammation reactions. Thus, long-term biosafety assessment will be performed in future studies to monitor ion bioelimination and chronic inflammation for full biosafety evaluation, facilitating further clinical translation of the CGO NF.

### 2.7 In vitro antibacterial activity of CGO NF

**Fig. 4:**
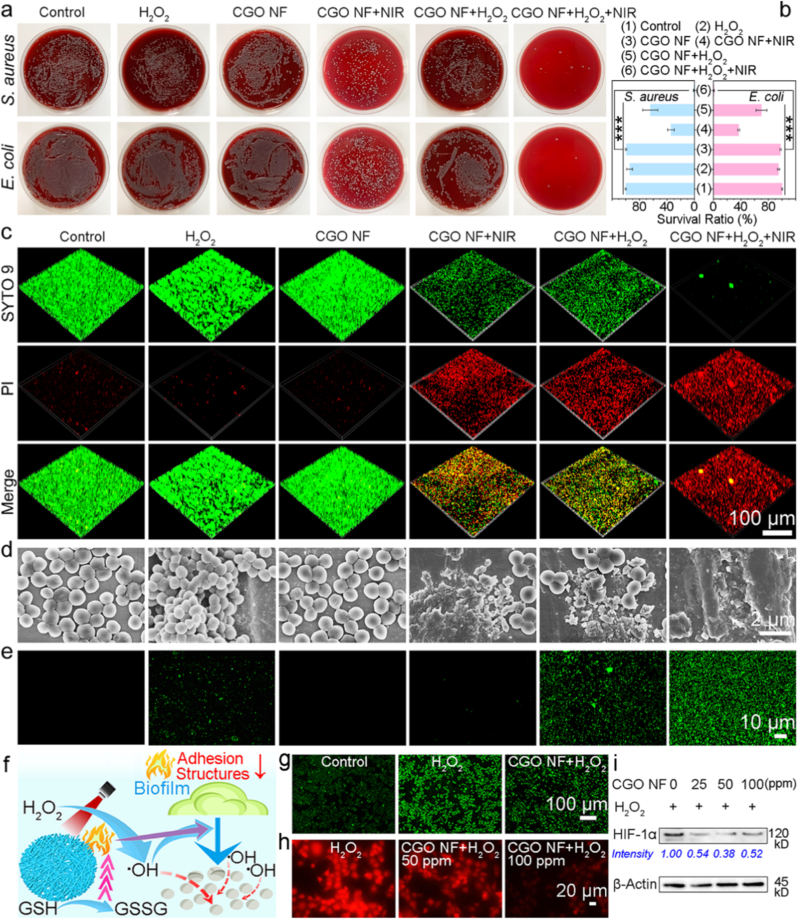
In vitro antibacterial activity and intracellular ROS elimination capability. (a) Photos and (b) quantitative analysis of bacterial colonies formed by *S. aureus* and *E. coli* planktonic bacteria treated with the indicated groups. (c) Live/dead staining and (d) SEM images of *S. aureus* biofilms on the titanium metal plate surfaces after indicated treatments. (e) Fluorescence images of ROS in *S. aureus* bacteria following indicated treatments. (f) A possible mechanism for the efficient antibacterial and antibiofilm activity of the CGO NF in vitro. (g) Fluorescence images of ROS in 3T3 cells after indicated treatments. (h) O_2_ generation in 3T3 cells using [Ru(dpp)_3_]Cl_2_ as a probe after indicated treatments. (i) Western blot of HIF-1α expression level in 3T3 cells after incubation with different concentrations of CGO NF in the presence of H_2_O_2_ under hypoxic conditions.

Encouraged by the superior photothermal performance and POD-like activity of the CGO NF under acidic conditions, we further assessed its antiplanktonic bacterial ability in vitro. Prior to that, the ability of the CGO NF to adsorb bacteria, possibly arising from its unique porous network structure, was observed (Fig. S37), beneficial to its antibacterial activity. After then, the in vitro photothermal antibacterial activity of the CGO NF was determined through the spread plate method. As shown in Fig. S38, upon 808 nm NIR laser irradiation (0.5 W/cm^2^) in the presence of the CGO NF, the bacterial survival ratio of both *S. aureus* and *E. coli* sharply declines within just 1 min, and continuingly decreases to 0.11% and 0.1%, respectively, with the irradiation time extended to 10 min. As a control, over 97% bacteria are still alive after 10 min of NIR laser irradiation alone. This validates the CGO NF has prominent photothermal antibacterial effect in vitro when exposed to NIR laser irradiation. Considering such prominent photothermal antibacterial effect of the CGO NF, we wonder whether CDT from its POD-like activity could aid PTT against bacteria to circumvent thermal damage to nearby normal tissues. Thereby, next, the antiplanktonic bacterial efficacy of different treatments was evaluated with the power density and time of NIR laser irradiation decreased to 0.3 W/cm^2^ and 5 min, respectively. Note that bacteria generally have weakly acidic microenvironments, and H_2_O_2_ was added to simulate the IMEs. It is evident from Fig. 4a and b that separate H_2_O_2_ or CGO NF has no noticeable influence on bacterial viability. In contrast, the viability of bacteria treated by NIR laser irradiation or H_2_O_2_ in the presence of the CGO NF exhibits a moderate decrease, suggesting a certain degree of PTT or CDT antibacterial effect of the CGO NF under these selected conditions. Remarkably, the CGO NF + H_2_O_2_ + NIR group presents only around 1% bacterial survival ratio for the two bacteria with almost disappearance of colonies in agar plates. It is the synergistic PTT and CDT effect of the CGO NF that facilitates such a significant decrease in bacterial viability. Simultaneously, this synergistic effect decreases the requisite temperature and time of PTT to ensure biosafety.

It has been suggested previously that once an infected wound progresses to a chronic state, the formation of a self-protective biofilm would bring about strengthened antibiotic resistance, immune evasion and persistent intractable infection, thus failing the wound infection therapy and healing [52]. Additionally, clinical investigations have demonstrated that biofilm infection is frequently induced by *S. aureus* [53]. Therefore, the antibiofilm efficacy of the CGO NF was further evaluated using the *S. aureus* biofilm. The typical live/dead staining of biofilms after different treatments was first carried out. As visualized in Fig. 4c, the Control, H_2_O_2_ and CGO NF groups display intact biofilms with bright green fluorescence (SYTO 9, indicating live bacteria) and negligible red fluorescence (propidium iodide (PI), indicating dead bacteria). In comparison, both the CGO NF + NIR and CGO NF + H_2_O_2_ groups exhibit dispersed and disrupted biofilms with higher red fluorescence, implying efficient PTT or CDT antibiofilm efficacy of the CGO NF. Excitingly, in the CGO NF + H_2_O_2_ + NIR group, nearly the whole biofilm presents red fluorescence, illustrating the best antibiofilm efficacy due to the combined PTT/CDT of the CGO NF, aligning with the previous result from the antiplanktonic bacterial experiment. Such severe damage to the biofilm is also testified by the SEM result (Fig. 4d).

To elucidate the underlying antibacterial and antibiofilm mechanism of the CGO NF, the level of ROS generation within bacteria following various treatments was measured as well (Fig. 4e; Fig. S39). No distinct fluorescence is observed in the Control, CGO NF, and CGO NF + NIR groups, indicating no pronounced ROS generation. Comparatively, the H_2_O_2_ group presents weak green ROS fluorescence, while the CGO NF + H_2_O_2_ group displays higher ROS fluorescence, primarily ascribed to the produced •OH by POD-like activity of the CGO NF. Apparently, the CGO NF + H_2_O_2_ + NIR group has the most significant ROS fluorescence, suggesting PTT could promote ROS generation in bacteria. Based on these findings and previous reports, a possible antibacterial and antibiofilm mechanism of the CGO NF is proposed (Fig. 4f). First, as a wound dressing, the CGO NF exhibits a unique porous network structure with large numbers of exposed active sites, compared to nanozymes integrated with hydrogels, microneedles, or fibers, conductive to capture bacteria and catalyze H_2_O_2_ to •OH [54]. Second, apart from direct killing of bacteria, the heat generated by PTT not only inhibits bacterial adhesion [55], but also disrupts biofilm and bacterial membrane structures [56]. Third, •OH generated via the POD-like activity of the CGO NF could penetrate the membrane, destroying nucleic acids and proteins [56], so as to assist photothermal killing of bacteria. Besides, the CGO NF possessing GSH depletion and heat production ability could boost the •OH generation.

### 2.8 Intracellular ROS elimination capability

Given the enzyme activity of the CGO NF self-switches to CAT-like activity under neutral conditions, its ROS elimination capability at the cellular level was further explored. To induce oxidative stress in cells, H_2_O_2_ was utilized. As depicted in Fig. 4g and Fig. S40, bright green ROS fluorescence is found in cells treated with H_2_O_2_, while the significantly weakened ROS fluorescence is detected after the addition of the CGO NF. Meanwhile, the red fluorescence of O_2_ probe [Ru(dpp)_3_]Cl_2_ is quenched by the generated O_2_ after treatment with the CGO NF in the presence of H_2_O_2_ (Fig. 4h; Fig. S41). Similarly, western blot analysis shows that, in the presence of H_2_O_2_, the expression level of HIF-1α is notably down-regulated after incubation with the CGO NF compared to that without the CGO NF (Fig. 4i), suggesting the CGO NF could relieve hypoxia via its CAT-like activity. Consequently, all these results certify the satisfactory intracellular ROS elimination capability of the CGO NF.

### 2.9 Intracellular anti-inflammatory and wound repair activity of CGO NF

**Fig. 5:**
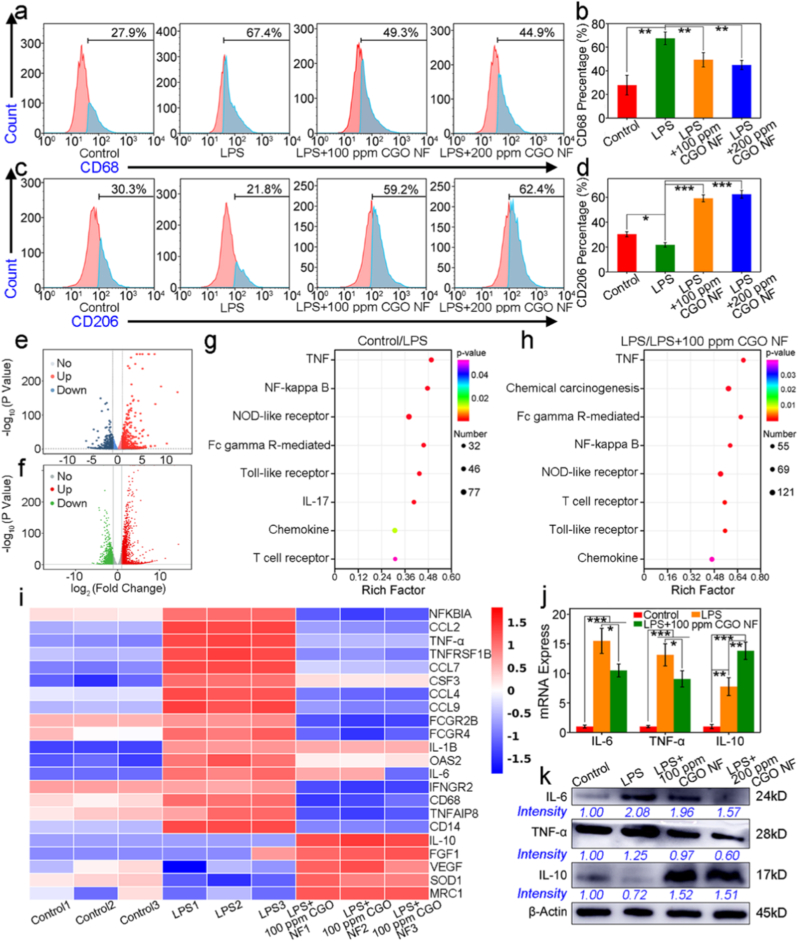
Intracellular anti-inflammatory activity of the CGO NF. Flow cytometric analysis for the expressions of (a,b) CD68 and (c,d) CD206 in Raw 264.7 cells after different treatments: (1) Control, (2) LPS, (3) LPS + 100 ppm CGO NF, and (4) LPS + 200 ppm CGO NF. Differential gene distributions volcano plots of (e) Control and LPS groups, and (f) LPS and LPS + 100 ppm CGO NF groups. KEGG enrichment analysis scatter diagrams of (g) Control and LPS groups, and (h) LPS and LPS + 100 ppm CGO NF groups. (i) Heat map assessing gene expressions in the pathways related to immune and inflammation. (j) qRT-PCR and (k) western blots evaluating the expressions of IL-6, TNF-α, and IL-10 in Raw 264.7 cells following indicated treatments.

After clarifying the intracellular ROS elimination capacities of this CGO NF under neutral conditions, it is reasonable to predict that the CGO NF should have the anti-inflammatory activity, as the elimination of ROS would initiate the polarization of macrophages from M1 to M2 phenotype [57]. It has been proven that M2 phenotype macrophages play a pivotal role in anti-inflammation and wound repair [58]. To validate the ability of the CGO NF to regulate macrophage polarization, lipopolysaccharide (LPS) was employed to stimulate Raw 264.7 cells to switch into M1 macrophages. As shown in Fig. 5a–d, as expected, LPS effectively induces the differentiation of Raw 264.7 cells into M1 macrophages. Remarkably, following treatment with the CGO NF (200 ppm), the expression of CD68, a marker of M1 macrophages, decreases significantly from 67.4% to 44.9%, while the expression of CD206, a marker of M2 macrophages, increases markedly from 21.8% to 62.4%. This demonstrates a pronounced increase in the ratio of M2 macrophages and a distinct decrease in the ratio of M1 macrophages, manifesting the phenotypic polarization of macrophages from M1 to M2 by the CGO NF.

It is noted that most of currently reported RNA sequencing assays in nanoagents-enabled wound infection therapy and healing focus on exploring the molecular mechanism underlying either the antibacterial effect or the macrophage polarization [57,58]. In this study, RNA sequencing was conducted to ascertain the intrinsic mechanism of anti-inflammatory and wound repair activity aroused by M2 macrophage polarization (Table S2). First, volcano plots in Fig. 5e exhibit that, when compared to the Control group, 2862 differentially expressed genes (DEGs) are upregulated, whereas 2523 DEGs are downregulated in the LPS group, indicating an evident variation in gene expression after LPS stimulation. Further Kyoto Encyclopedia of Genes and Genomes (KEGG) analysis reveals that signaling pathways related to immunity and inflammation are greatly affected by LPS stimulation, such as the tumor necrosis factor (TNF), nuclear factor (NF)-kappa B, and Fc gamma receptor (R)-mediated signaling pathways, with higher rich factors and lower p-values (Fig. 5g). Meanwhile, the heatmap of DEGs vividly displays the expressions of pro-inflammatory genes are noticeably upregulated (Fig. 5i). These results confirm that LPS successfully stimulated the transformation of macrophage to the M1 pro-inflammatory phenotype. On the other hand, intriguingly, 1976 upregulated DEGs and 1777 downregulated DEGs are also identified in the volcano plots between the LPS group and LPS + 100 ppm CGO NF group (Fig. 5f). Similar to the case between the Control group and the LPS group, KEGG pathway analysis between the LPS group and LPS + 100 ppm CGO NF group highlights that signaling pathways associated with immunity and inflammation, mainly including the TNF, Fc gamma R-mediated, and chemokine signaling pathways, are activated as well (Fig. 5h). This illustrates the CGO NF exerts a distinct impact on the immune and inflammatory response. Further heatmap analysis finds that, compared with the LPS group, pro-inflammatory genes, like TNF-α, tumor necrosis factor-α-induced protein 8 (TNFAIP8), nuclear factor-kappa-B-inhibitor alpha (NFKBIA), interleukin-6 (IL-6), C-C motif chemokine ligand 2 (CCL2), CCL4, Fc gamma R IIB (FCGR2B), FCGR4 and interferon gamma R 2 (IFNGR2), are significantly downregulated, while the anti-inflammatory genes, involving IL-10, superoxide dismutase 1 (SOD1) and mannose R C type 1 (MRC1), are substantially upregulated in the LPS + 100 ppm CGO NF group (Fig. 5i). More importantly, vascular endothelial growth factor (VEGF) and fibroblast growth factor 1 (FGF1) genes that accelerate cell proliferation, cell migration and angiogenesis for wound healing are found notably upregulated in the LPS + 100 ppm CGO NF group. Such anti-inflammatory activity of the CGO NF was further verified through qRT-PCR and western blots of IL-6, IL-10 and TNF-α genes and proteins, respectively (Fig. 5j and k). Altogether, the above evidence suggests that the CGO NF could effectively alleviate inflammation and promote wound repair via regulating immunity- and inflammation-related signaling pathways.

**Fig. 6:**
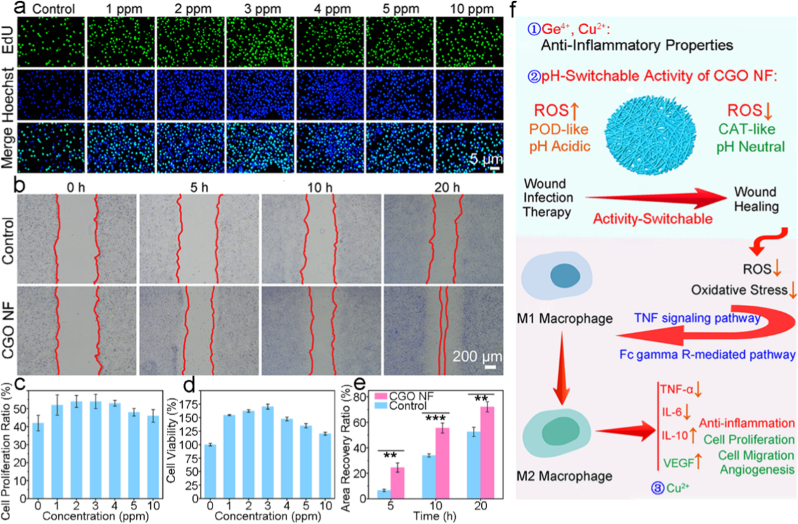
Intracellular wound repair activity and schematic illustration for intracellular anti-inflammatory and wound repair activity of the CGO NF. Representative images of (a) cell proliferation analyzed by EdU incorporation assay after treatment with the CGO NF at various concentrations and (b) the migration of 3T3 cells at different time points post treatment with the CGO NF. (c) Cell proliferation ratio of 3T3 cells after incubation with different concentrations of the CGO NF by EdU incorporation assay. (d) Relative cell viability of 3T3 cells after incubation with different concentrations of the CGO NF by CCK-8 assay. (e) Quantitative analysis of migration of 3T3 cells after treatment with the CGO NF for different time. (f) Schematic illustration for intracellular anti-inflammatory and wound repair activity of the CGO NF.

It has been widely recognized that Cu^2+^ could stimulate cell proliferation, cell migration, angiogenesis and collagen deposition that are essential for wound healing [59]. Considering the CGO NF dressing releases a very low dosage of Cu^2+^ during the wound healing process, the cell proliferation and cell migration capability of the CGO NF were subsequently studied to appraise its wound repair activity. Unsurprisingly, the evidently increased cell proliferation, relative to the Control group, is detected by 5-ethynyl-2′-deoxyuridine (EdU) incorporation assay after treatment of the CGO NF at low concentrations below 10 ppm (Fig. 6a–c). Moreover, the cell proliferation ratio increases with the CGO NF concentration and peaks at 3 ppm, presenting a similar tendency to the result of CCK-8 assay (Fig. 6d). Consistently, treatment with the CGO NF at 3 ppm facilitates the cell migration, closing the scratch gap of 72.1% in comparison with 52.6% in the control group at 20 h (Fig. 6b–e).

Based on above results and cognitions, as illustrated in Fig. 6f, we believe that such anti-inflammatory and wound repair activity of the CGO NF mainly comes from its enzyme activity that automatically switches into CAT-like activity under the neutral microenvironments. With this pH-switchable activity, the CGO NF could rapidly eradicate ROS, thereby regulating immunity- and inflammation-related signaling pathways and promoting the polarization of macrophages to M2 anti-inflammatory phenotype. M2 macrophage polarization modulates inflammatory and active factors to relieve inflammation and accelerate wound repair. In addition, Cu^2+^ released from the CGO NF can also accelerate wound repair. Beyond that, importantly, Ge^4+^ and Cu^2+^ themselves from the CGO NF possibly possess anti-inflammatory properties according to prior reports [29,30].

### 2.10 Wound infection therapy and healing of CGO NF

**Fig. 7:**
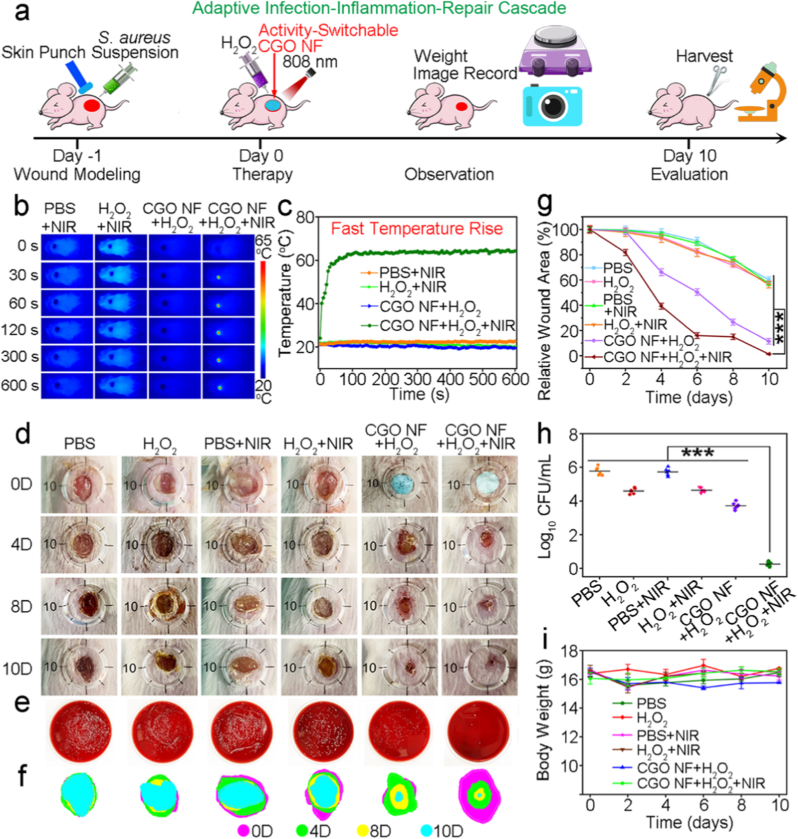
Wound infection therapy and healing of the CGO NF. (a) Schematic diagram for the model building of *S. aureus* infected wound and the therapy process with the CGO NF. (b) IR thermal imaging of mice with infected wounds treated with (1) PBS + NIR, (2) H_2_O_2_ + NIR, (3) CGO NF + H_2_O_2_, and (4) CGO NF + H_2_O_2_ + NIR groups and (c) corresponding temperature change curves of the infected sites in mice with irradiation time. Representative photos of (d) infected wounds and (e) bacterial colonies formed in wounds before and 4, 8, and 10 days after various treatments: (1) PBS, (2) H_2_O_2_, (3) PBS + NIR, (4) H_2_O_2_ + NIR, (5) CGO NF + H_2_O_2_, and (6) CGO NF + H_2_O_2_ + NIR. (f) Schematic diagram of time-evolved wound areas for each group. Corresponding quantitative analysis of (g) the relative wound areas and (h) the bacterial numbers in wounds in different treatments. (i) Body weight changes of the mice in the different treatment groups during the wound healing.

Inspired by such comprehensive water/blood absorbing, wet tissue adhesion, antibacterial, anti-inflammatory and wound repair activity, together with favorable biocompatibility of activity-switchable CGO NF presented above, we attempted to apply this flexible NF as a wound dressing for realizing adaptive infection-inflammation-repair cascade in wound infection therapy and healing. To this end, mouse skin full-thickness round-shaped wounds infected by *S. aureus* was established (Fig. S42). These infected wounds were randomly assigned into six groups (*n* = 6) and underwent following treatments: PBS, H_2_O_2_, PBS + NIR (808 nm, 0.3 W/cm^2^), H_2_O_2_ + NIR, CGO NF + H_2_O_2_ and CGO NF + H_2_O_2_ + NIR. The entire wound infection therapy and healing procedure is depicted in Fig. 7a. The IR thermal imaging recording wound temperature alteration is presented in Fig. 7b. It can be observed that upon exposure to NIR laser irradiation, the wound temperature in the CGO NF + H_2_O_2_ + NIR group very quickly elevates from 24.0 to 40.1 ^o^C within just 5 s, and then plateaus at around 64 ^o^C. By contrast, the temperature in other groups displays no significant change (Fig. 7c). Of note, such a fast temperature rise, combined with precisely tuned irradiation time, contributes to localized heat focusing. Specifically, the NIR laser power density used in this work is 0.3 W/cm^2^ that is below the safe power density threshold (∼0.33 W/cm^2^) for the 808 nm NIR laser [60]. This will strengthen the efficacy of photothermal antibacterial therapy while minimizing side effects to surrounding healthy skin tissues.

Then, sequential photos of representative infected wounds on mice from each treatment group over 10 days were captured. As shown in Fig. 7d and S43, all wounds heal progressively with time. Remarkably, the wound in the CGO NF + H_2_O_2_ + NIR group represents far faster healing rate than those in the other five groups. Notice that the CGO NF + H_2_O_2_ group exhibits a moderate decrease in wound area compared to the other groups, likely due to the limited antibacterial effect of CDT that, as discussed above, could assist PTT to ensure biosafety. Meanwhile, varying degrees of inflammation in the process of the wound healing are observed for all groups from macroscopic views, and CGO NF + H_2_O_2_ + NIR group has the lightest inflammation. Further evolution diagram and corresponding quantitative analysis of wound areas (Fig. 7f and g; Fig. S44) reveal that the wound in the CGO NF + H_2_O_2_ + NIR group is almost healed with the wound area decreasing considerably to only 1.3% (98.7% wound closure) by day 10, whereas 60.2%, 57.5%, 57.1%, 56.3% and 11.4% of the wound areas remain in the PBS, H_2_O_2_, PBS + NIR, H_2_O_2_ + NIR, and CGO NF + H_2_O_2_ groups, respectively. Besides, residual bacteria in wounds were appraised on day 10 post treatments. As illustrated in Fig. 7e–h, few bacterial colonies are observed in the CGO NF + H_2_O_2_ + NIR group, corroborating the superior in vivo antibacterial efficacy of this treatment. Also, the CGO NF + H_2_O_2_ group shows a modest reduction in the number of bacterial colonies, ascribed to the antibacterial effect of CDT. In contrast, a significant number of bacterial colonies are present in the other groups, which inhibits the wound healing process. Further, both the H&E staining result of major organs from the CGO NF + H_2_O_2_ + NIR group on day 10 (Fig. S45) and the stable body weight of mice during the wound healing (Fig. 7i) underscore the high biosafety of the CGO NF + H_2_O_2_ + NIR treatment. These findings authenticate the superior and safe wound infection therapy and healing capability of NIR laser-activated CGO NF with H_2_O_2_.

**Fig. 8:**
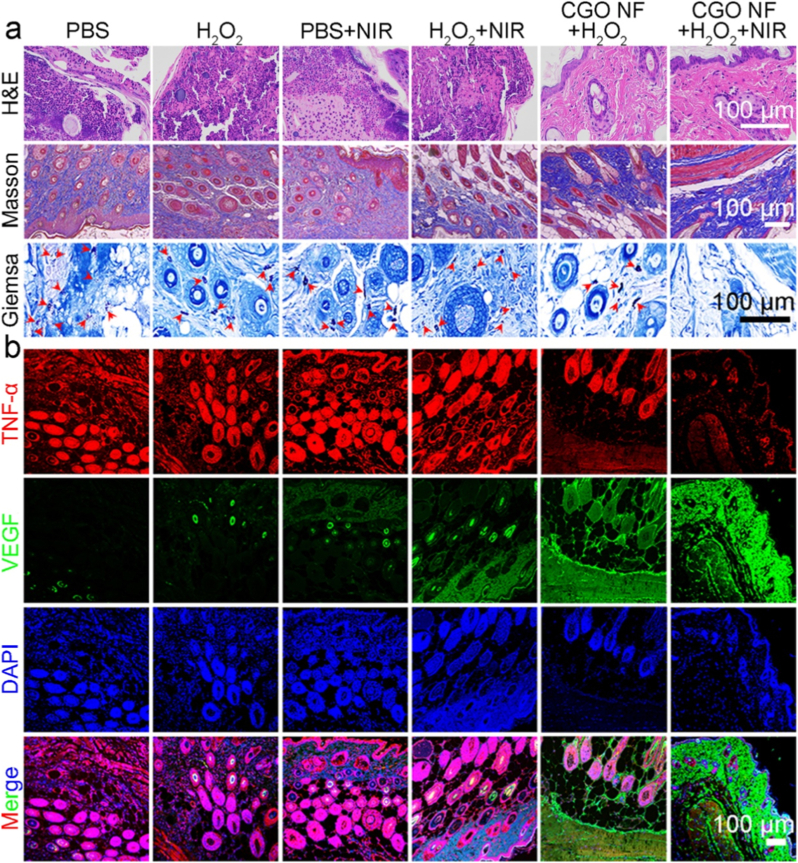
Histopathological and immunofluorescence assays of infected wounds subjected to varying treatments. (a) H&E, Masson and Giemsa staining images of **wound bed tissues** 10 days after various treatments. The red arrows specifically designate the presence of infectious bacteria. (b) Immunofluorescence staining images of TNF-α and VEGF in **wound bed tissues** 10 days following different treatments. (For interpretation of the references to color in this figure legend, the reader is referred to the Web version of this article.)

More importantly, to comprehensively evaluate the wound healing quality, histological analysis of the wound bed tissues 10 days after treatments was performed using H&E, Masson, and Giemsa staining (Fig. 8a; Fig. S46). As seen in the H&E staining images, the relatively intact skin structures with restored epidermises and less inflammatory cells are visible in both the CGO NF + H_2_O_2_ and CGO NF + H_2_O_2_ + NIR groups. On the contrary, evident epidermis defects and extensive inflammatory cell infiltration could be found in the other groups. Meanwhile, from the Masson staining images, the CGO NF + H_2_O_2_ + NIR group displays more continuous and pronounced collagen deposition compared to all other groups, implying elevated collagen content. As the collagen is critical to granulation tissue formation and skin regeneration, such increased collagen content observed after CGO NF + H_2_O_2_ + NIR treatment efficiently facilitates wound repair. Moreover, it is observed from the Giemsa staining images that few and some bacteria remain in the wounds after treatments with CGO NF + H_2_O_2_ + NIR and CGO NF + H_2_O_2_ groups, respectively. However, there still exist many bacteria in the wounds of the other groups. These results agree well with the data in Fig. 7, demonstrating NIR laser-activated CGO NF with H_2_O_2_ was the most effective in wound infection therapy and healing resulting from its superior antibacterial effect, reduced inflammatory cell infiltration, promoted re-epithelialization and enhanced collagen deposition.

Immunofluorescence staining was further performed to examine the expression levels of dihydroethidium (DHE), TNF-α and VEGF. Initially, the ROS levels in wound bed tissues at 5 days post treatments were appraised by DHE staining (Fig. S47). As expected, the ROS levels of CGO NF + H_2_O_2_ and CGO NF + H_2_O_2_ + NIR groups decrease significantly compared to those of other groups, suggesting the CGO NF could effectively eliminate ROS in wound bed tissues. This offers convincing evidence that the CGO NF exerts CAT-like activity during the wound-healing stage to alleviate inflammation and promote wound repair. The levels of TNF-α and VEGF were further explored 10 days after treatments. As can be clearly seen from Fig. 8b, compared to the other groups, the CGO NF + H_2_O_2_ + NIR group noticeably decreases the expression level of TNF-α, and at the same time increases the expression of VEGF, indicating the highest degree of anti-inflammatory and angiogenesis ability of NIR laser-activated CGO NF with H_2_O_2_ among all treatment groups. These results certify that the CGO NF expedites the transition of macrophages from the M1 to the M2 phenotype in vivo to regulate inflammatory and active factors for anti-inflammation and wound repair. Such ability of the CGO NF for anti-inflammation and wound repair renders it a prospective candidate for practical application in wound healing.

## 3 Conclusion

In summary, for the first time, our study has successfully designed and developed a freestanding pure CGO NF consisting of nanowires via a straightforward approach. Ascribed to its flexibility, wet tissue adhesion properties, as well as water/blood absorbing capability, such a NF is demonstrated to act as a wound dressing. Besides, extraordinarily excellent photothermal performance due to its high NIR absorbance, and GSH depletion-enhanced ROS generation from its POD-like activity in acidic IMEs enable the CGO NF to possess a superior antibacterial/antibiofilm effect for PTT/CDT synergistic therapy of wound infection. Interestingly, following the infection eradication, the acidic IMEs switch into neutral microenvironments, activating the enzyme activity of the CGO NF to automatically and continuously switch into CAT-like activity to remove excess ROS. This induces M1 macrophage polarization towards M2 macrophages, and signaling pathways in immunity and inflammation such as the TNF, Fc gamma R-mediated, and chemokine signaling pathways, are activated. M2 macrophage polarization leads to anti-inflammation and wound repair. It is noteworthy that Ge^4+^ and Cu^2+^ themselves in CGO possibly own anti-inflammatory ability and Cu^2+^ could also stimulate wound repair. Simultaneously, the longer and entangled nanowire structure of the CGO NF inclined to be detained in the wound site minimizes its toxicity to normal tissues. Hence, a safe, efficacious activity-switchable photothermal NF dressing that could intelligently respond to the microenvironments change has been highlighted to ultimately achieve adaptive infection-inflammation-repair cascade in wound infection therapy and healing. This work not only provides a brand-new possibility of low-dimensional inorganic nanomaterials in microenvironments-sensitive biomedical soft materials, but also a novel pathway to develop activity-switchable nanozymes for other redox-related pathological contexts, such as refractory diabetic wounds and infectious diseases. However, future research should further clarify the long-term biosafety, catalytic stability and systemic immune responses of CGO NFs to meet clinical regulatory standards for wound dressings, and reduce costs via large-scale production to maximize their clinical translation potential.

## CRediT authorship contribution statement

**Wenlong Zhang:** Conceptualization, Funding acquisition, Investigation, Methodology, Software, Writing – original draft. **Yilin Yuan:** Conceptualization, Formal analysis, Methodology, Software. **Jiaqi Liu:** Data curation, Formal analysis. **Jiang Du:** Data curation, Formal analysis. **Xinxin Zhao:** Data curation, Formal analysis. **Zhen Jin:** Data curation, Formal analysis. **Xianglong Zhu:** Validation, Visualization. **Xiaojuan Huang:** Project administration, Resources. **Dapeng Wu:** Conceptualization, Funding acquisition, Resources, Supervision, Writing – review & editing. **Junqing Hu:** Conceptualization, Funding acquisition, Resources, Supervision, Writing – review & editing.

## Declaration of competing interest

The authors declare that they have no known competing financial interests or personal relationships that could have appeared to influence the work reported in this paper.

## Data Availability

Data will be made available on request.
