# Supplementary material for: Immuno-modulatory nanozyme film with self-switchable activity for adaptive infection-inflammation-repair cascade in wound infection therapy and healing
Source: Mater Today Bio. 2026 Jul 15;39:103465. doi: 10.1016/j.mtbio.2026.103465 (PMC13400452; doi:10.1016/j.mtbio.2026.103465)
Supplement: Multimedia component 1 [file mmc1.docx]

Supporting Information

**Immuno-Modulatory Nanozyme Film with Self-Switchable Activity for Adaptive Infection-Inflammation-Repair Cascade in Wound Infection Therapy and Healing**

Wenlong Zhang,^a,1,*^ Yilin Yuan,^a,1^ Jiaqi Liu,^a^ Jiang Du,^a^ Xinxin Zhao,^e^ Zhen Jin,^a^ Xianglong Zhu,^a^ Xiaojuan Huang,^d^ Dapeng Wu,^c,*^ Junqing Hu^b,*^

Materials and Methods

*Materials*: GeO_2_, ethylenediamine (En), Cu(CH_3_COO)_2_•H_2_O, glutathione (GSH), dimethyl sulfoxide (DMSO), 3,3’,5,5’-tetramethylbenzidine (TMB), 5,5-Dimethyl-1-pyrroline N-oxide (DMPO) were procured from Aladdin Chemistry Co., Ltd. H_2_O_2_ solution (w/w: 30%) was supplied by Sinopharm Chemical Reagent Co., Ltd. 5,5’-dithiobis (2-nitrobenzoic acid) (DTNB) was sourced from Macklin Biochemical Co., Ltd. 2’,7’-dichlorodihydrofluorescein diacetate (DCFH-DA), lipopolysaccharide (LPS) were purchased from Sigma-Aldrich Co., Ltd. All chemicals were of analytic grade and used as received without further purification.

*Synthesis of the CuGeO_3_ (CGO) Nanozyme Film (NF)*: GeO_2_ (0.31 g) and En (150 μL) were dissolved in 27 mL of deionized water, resulting in a homogeneous transparent solution. Subsequently, 15 mL of aqueous solution containing 0.60 g of Cu(CH_3_COO)_2_•H_2_O was added dropwise into the above solution under vigorous stirring to form a blue suspension. With continuous stirring for another 10 min, this suspension was transferred into a Teflon-lined stainless-steel autoclave (60 mL of capacity) and heated at 200 ^o^C for 96 h. After the reaction, the product of light blue sediment was collected by centrifugation and washed with deionized water and ethanol. Then the sediment was redispersed in deionized water and vacuum filtrated to form a blue film. This film was dried in air at room temperature and peeled off from the substrate membrane (a micropore filter membrane (Millipore), pore size: 0.22 µm, diameter: 50 mm) of the filter to obtain the final freestanding CGO NF. For comparison, the products were also created with reaction temperature of 200 ^o^C for different reaction time (24, 48 and 72 h), as well as reaction time of 24 h at varying reaction temperature (160 and 180 ^o^C).

*Characterization*: Scanning electron microscopy (SEM) images were taken on a S-4800 scanning electron microscope (Hitachi). Nitrogen adsorption-desorption isotherms were carried out with an ASAP 2420 nitrogen adsorption apparatus (Micrometrics Instruments). Pore size distribution plots were achieved by the Barrett-Joyner-Halenda (BJH) method. Transmission electron microscopy (TEM) images were observed with a JEM-2100F transmission electron microscope (JEOL). X-ray diffraction (XRD) measurement was acquired by a D/max-2550 PC X-ray diffractometer (Rigaku). Ultraviolet-visible-near-infrared (UV-Vis-NIR) absorption spectra were performed on a UV-Vis 1901 spectrophotometer (Phoenix). UV-Vis-NIR diffuse reflectance spectrum was measured using a UV-Vis-NIR spectrophotometer (Shimadzu UV-3600). The contact angle was tested by a JY-82B Kruss DSA contact angle meter. Fourier transform infrared (FTIR) spectrum was collected by an IR spectrophotometer (NICOLET NEXUS670). The electron spin resonance (ESR) spectrum measurements were conducted by a Bruker EMXnano ESR spectrometer. The dissolved oxygen concentration was determined by a JPB-607A dissolved oxygen meter (Leici). Concentrations of Cu and Ge were measured using a Leeman Laboratories Prodigy high dispersion inductively coupled plasma atomic emission spectroscopy (ICP-AES).

*Density Functional Theory (DFT) Calculations*: The calculations were carried out in the framework of the DFT with the projector augmented plane-wave method, as implemented in the Vienna ab initio simulation package [1]. The generalized gradient approximation proposed by Perdew, Burke and Ernzerhof was selected for the exchange-correlation potential [2]. The cut-off energy for plane wave was set to 500 eV. The energy criterion was set to 10-5 eV in iterative solution of the Kohn-Sham equation. The Brillouin zone integration was performed using a 7×4×11 k-mesh. All the structures were relaxed until the residual forces on the atoms declined to less than 0.02 eV/Å. The energy band and projected density of state were calculated. At the same time, Gaussian 09 software was used to optimize the TDDFT of the molecule using the B3LYP/def2TZVP functional basis set [3]. The resulting output file was then combined with the Multiwfn program to draw the UV-Vis-NIR absorption spectrum [4].

*Photothermal Performance of the CGO NF*: Initially, the temperature of the CGO NF under 808 nm NIR laser irradiation (Shanghai Xilong Optoelectronic Technology CO., Ltd., China) at different power densities (0.1, 0.2, 0.3, 0.5, 0.7 and 1.0 W/cm^2^), and with a working distance of 20 cm and a spot area of ~0.38 cm^2^ for 5 min was recorded. After that, the photothermal stability of the CGO NF was investigated using 60 on/off laser cycles. One cycle is that the CGO NF was irradiated by 808 nm NIR laser with a power density of 0.3 W/cm^2^ for 30 s and then the NIR laser was turned off for 30 s to cool the NF to room temperature. Then, the temperature of PBS (pH 7.4), PBS (pH 7.4) + CGO NF (a 6-mm-diameter round NF, without ultrasonic dispersion), PBS (pH 7.4) + Glucose (25 mM) + GSH (8 mM), PBS (pH 7.4) + Glucose (25 mM) + GSH (8 mM) + CGO NF, PBS (pH 5.5) + Glucose (25 mM) + GSH (8 mM), and PBS (pH 5.5) + Glucose (25 mM) + GSH (8 mM) + CGO NF under 808 nm NIR laser irradiation of 0.7 W/cm^2^ for 5 min was recorded. The temperature was monitored and imaged concurrently with a FLIR A300 thermal imaging camera.

*Deep-Seated Photothermal Effect Evaluation*: A CGO NF covered with a pig skin (thickness: 3.8 mm), the CGO NF, and the pig skin were exposed to 808 nm laser irradiation of 1.0 W/cm^2^ for 5 min, and the temperature was monitored and imaged concurrently with the thermal imaging camera.

*GSH Depletion*: To test the GSH depletion, the CGO NF (100 ppm, ultrasonic dispersion) was co-incubated with GSH aqueous solution (0.4 mM), DTNB in DMSO solution (100 mM) and phosphate buffered saline (PBS, pH 7.4) with or without glucose (25 mM) for varying reaction time (0, 1, 2, 3, 4, 5, 6 and 7 h). Eventually, the supernatant was collected by centrifugation and detected via a UV-Vis spectrophotometer. For comparison, the GSH depletion by commercial bulk GeO_2_ was also tested under the same conditions without glucose.

*POD-like Activity and Kinetic Assay*: The POD-like activity of the CGO NF was detected using TMB as the substrate in the presence of H_2_O_2_. Briefly, the CGO NF (100 ppm, ultrasonic dispersion), H_2_O_2_ (10 mM), TMB (20 mM) and PBS solution (pH 5.5) with or without glucose (25 mM) and GSH (8 mM) were mixed. After a certain reaction time (0, 1, 2, 3, 4, 5, 6, 7 and 8 min), the absorbance of the color reactions was measured by a UV-Vis spectrophotometer. For comparison, the POD-like activity of commercial bulk GeO_2_ was detected as well under the same conditions without glucose and GSH.

To detect the pH and temperature dependence of POD-like activity, the CGO NF (100 ppm, ultrasonic dispersion), H_2_O_2_ (10 mM), TMB (20 mM) and PBS solution (pH 5.5, 6.5 and 7.4) were mixed with or without 808 nm NIR laser irradiation (1.0 W/cm^2^) for 3 min. Furthermore, the absorbance of the color reactions was tested by a UV-Vis spectrophotometer.

Meanwhile, the steady-state kinetic assay was studied by employing different concentrations of H_2_O_2_ (final concentrations: 10, 20, 40, 80 and 160 mM) while maintaining other conditions the same. The absorbance variations of the solutions at 652 nm were recorded by UV-Vis spectra. Next, the absorbance could be converted into the oxidized TMB or generated •OH concentration via Beer-Lambert law:

*A* = *εbc* (1)

where *A* is the absorption value (*λ* = 652 nm), *ε* is a constant (39000 M^-1^ cm^-1^) for the molar absorption coefficient of oxidized TMB, *b* is the optical distance (1 cm), and *c* is the oxidized TMB concentration.

The Michaelis-Menten kinetic curve of the CGO NF was obtained through plotting the initial velocities of reaction against H_2_O_2_ concentrations according to the equation (2):

$v_{0}=\frac{V_{max}\times[H_{2}O_{2}]}{K_{m}+[H_{2}O_{2}]}$ (2)

where *v_0_* is the initial velocity of the reaction, *V_max_* is the maximum velocity of the reaction, [H_2_O_2_] is the H_2_O_2_ concentration in the solution, *K_m_* is the Michaelis-Menten constant. Then, the values of *K_m_* and *V_max_* were calculated according to Lineweaver-Burk plotting (3):

$\frac{1}{v_{0}}=\frac{K_{m}}{V_{max}}\times\frac{1}{[H_{2}O_{2}]}+\frac{1}{V_{max}}$ (3)

For ESR measurement of the CGO NF, the CGO NF (100 ppm, ultrasonic dispersion) was added into PBS solution (pH 5.5) containing H_2_O_2_ (1.0 mM), utilizing DMPO as the trapping agent to capture •OH.

*CAT-like Activity*: The CAT-like activity of the CGO NF was evaluated by monitoring the generation of dissolved oxygen from the catalysis of H_2_O_2_. Shortly, the CGO NF (100 ppm, ultrasonic dispersion) was mixed with H_2_O_2_ (10 mM) in PBS solution (pH 5.5, 6.5 and 7.4) with or without glucose (25 mM), and the produced dissolved oxygen amount (mg/L) was recorded within 20 min by a dissolved oxygen meter. For comparison, the CAT-like activity of commercial bulk GeO_2_ was further evaluated under the same conditions without glucose.

*In Vitro Cytotoxicity Assay*: To appraise the cytotoxicity of the CGO NF, a standard Cell Counting Kit-8 (CCK-8; Dojindo Molecular Technologies, Inc.) assay was performed. Typically, HaCaT, 3T3, and human skin fibroblast (HSF) cells were seeded in 96-well plates at a density of 1 × 10^4^ cells/well. After 24 h of incubation, the cells were treated with the CGO NF dispersed in Dulbecco’s Modified Eagle’s Medium (DMEM) at varying concentrations (0, 20, 40, 80, 160, 320, 640 and 1280 ppm, ultrasonic dispersion) for 24 h. Subsequently, the cells were washed with PBS three times, and 100 μL of fresh culture medium containing 10% CCK-8 was added to each well. After 2 h of treatment, absorbance at 450 nm was measured by a microplate reader.

Also, to simulate the real interaction between the CGO NF and tissue, the cytotoxicity of the CGO NF on 3T3 cells was examined in a transwell insert system without ultrasonic dispersion.

*Hemolysis Assay*: Red blood cells were achieved from blood of healthy mice by centrifugation and then washed several times with PBS (pH 7.4). The diluted cell suspensions (10% in PBS) were incubated with the CGO NF at various concentrations (20, 40, 80, 160, 320, 640 and 1280 ppm, ultrasonic dispersion) for 6 h. The deionized water and PBS served as positive and negative controls. Afterward, the samples were centrifuged and the absorbance of the supernatants was measured at 540 nm with a microplate reader. The hemolysis ratio (%) was calculated using the following formula:

Hemolysis ratio (%) = $\frac{{OD}_{sample}-{OD}_{PBS}}{{OD}_{water}-{OD}_{PBS}}$ × 100% (4)

*In Vitro Antiplanktonic Bacteria*: The in vitro antiplanktonic bacteria of the CGO NF was visually observed through the spread plate method (SPM) to determine the number of colony forming units (CFU) on the plates. The gram-negative bacterium *Escherichia coli* (*E. coli*, ATCC 35218) and the gram-positive bacterium *Staphylococcus aureus* (*S. aureus*, ATCC 43300) were utilized for antibacterial tests.

These bacteria (*E. coli and S. aureus*) were incubated in Luria-Bertani broth medium (Oxoid) in a shaker at 37 °C for 12 h. Following incubation, a 6-mm-diameter round CGO NF (achieved by using a hole punch) in 100 μL of PBS without ultrasonic dispersion was mixed with 50 μL of 10^7^ CFU/mL bacterial suspension (*E. coli or S. aureus*) in 96-well plates. With or without the addition of 50 μL of H_2_O_2_ (10 μM), as well as either with or without 808 nm NIR laser irradiation (Shanghai Xilong Optoelectronic Technology CO., Ltd., China) with a power density of 0.3 W/cm^2^ and a spot area of ~12.6 cm^2^ for 5 min, they were thus divided into six groups: (1) Control, (2) H_2_O_2_, (3) CGO NF, (4) CGO NF + NIR, (5) CGO NF + H_2_O_2_, (6) CGO NF + H_2_O_2_ + NIR (For comparative analysis, bacterial suspension without any treatment was used as a control). Ultimately, all samples were diluted and the survival number of planktonic bacteria was counted by the SPM. The antibacterial survival ratio was calculated using the formula below:

Survival ratio (%) = $\frac{{CFU}_{sample}}{{CFU}_{control}}$ × 100% (5)

Additionally, following the same procedure and maintaining other conditions identical, 50 μL of 10^7^ CFU/mL bacteria (*E. coli and S. aureus*) exposed to 0.3 W/cm^2^ 808 nm NIR laser with a spot area of ~12.6 cm^2^ for different time (0, 1, 3, 5 and 10 min) after incubation with a 6-mm-diameter round CGO NF in 100 μL of PBS without ultrasonic dispersion were also investigated.

*In Vitro Antibiofilm*: Each medical titanium metal plate (10 × 10 × 1 mm^3^) immersed in 1 mL of 10^7^ CFU/mL bacterial suspension (*S. aureus*) was incubated statically in 24-well plates at 37 ^o^C overnight to form the biofilm on its surface. Each incubated titanium plate was lightly rinsed with PBS to remove nonadherent bacteria on the surface and placed into new 24-well plates containing a 6-mm-diameter round CGO NF in 500 μL of PBS without ultrasonic dispersion. Subsequently, after treatment with or without 250 μL of H_2_O_2_ (10 μM), as well as with or without irradiation by 0.3 W/cm^2^ 808 nm NIR laser with a spot area of ~12.6 cm^2^ for 5 min, they were divided into six groups: (1) Control, (2) H_2_O_2_, (3) CGO NF, (4) CGO NF + NIR, (5) CGO NF + H_2_O_2_, (6) CGO NF + H_2_O_2_ + NIR (For comparison, biofilm-coated titanium metal plate without any treatment functioned as the control group).

For evaluation of the antibiofilm efficiency by confocal laser scanning microscopy (CLSM), each plate was put into new 24-well plates and then stained with a live/dead BacLight bacterial viability kit (Invitrogen) involving SYTO 9 and propidium iodide (PI). After gently rinsing the residual probes with PBS, the stained biofilm was observed under a Nikon A1-Si CLSM (red and green fluorescence for PI and SYTO 9).

For evaluation of the antibiofilm efficiency by scanning electron microscopy (SEM), the plates were fixed with 2.5% glutaraldehyde solution at 4 °C for 4 h, then continuously dehydrated in 50%, 70%, 80%, 90%, 95% and 100% ethanol/water mixtures in new 24-well plates for 10 min, and eventually freeze-dried and sputtered with gold layer for SEM observation.

*Detection of Reactive Oxygen Species (ROS) in Bacteria*: The DCFH-DA probe was used to appraise ROS levels in bacteria. Differently treated *S. aureus* bacteria described as above experimental steps of in vitro antiplanktonic bacteria experiment were stained with DCFH-DA, and imaged using a Leica DMi8 fluorescence microscopy. The fluorescence in bacteria was quantified by Image-J software.

*Measurement of Intracellular ROS*: H_2_O_2_ was selected to induce the exogenous oxidative stress against 3T3 cells. 3T3 cells were seeded in a 6-well plate at a density of 1 × 10^5^ cells per well and incubated for 24 h. After treatment with H_2_O_2_ (200 μM) with or without the addition of a 6-mm-diameter round CGO NF in 100 μL of PBS without ultrasonic dispersion for 1 h, they were divided into three groups: (1) Control, (2) H_2_O_2_, and (3) CGO NF + H_2_O_2_. Subsequently, the culture medium was replaced by DCFH-DA probe, and fluorescence images were recorded by the fluorescence microscopy. The intracellular fluorescence was quantified with Image-J software.

*Evaluation of Intracellular O_2_ Generation*: Evaluation of intracellular O_2_ generation was performed in 3T3 cells incubated in hypoxic conditions after the CGO NF treatment in the presence of H_2_O_2_. Hypoxic conditions were achieved by incubation in hypoxia chamber with 1 % O_2_, 5 % CO_2_, and 94 % N_2_ for 4 h. Specifically, 3T3 cells were seeded in a 6-well plate at a density of 1 × 10^5^ cells per well and incubated for 24 h. After treating with different concentrations of CGO NF (0, 50 and 100 ppm, ultrasonic dispersion) in the presence of H_2_O_2_ (200 μM), 3T3 cells were incubated in hypoxic conditions for 4 h, followed by staining with [Ru(dpp)_3_]Cl_2_ (Sigma-Aldrich), and fluorescence images were recorded by the fluorescence microscope.

*Assessment of Hypoxic Degree by Western Blot:* 3T3 cells were incubated in hypoxic conditions after treatments with different concentrations of CGO NF (0, 25, 50 and 100 ppm, ultrasonic dispersion) in the presence of H_2_O_2_ (200 μM) for 4 h. Note that such hypoxic conditions were achieved by incubation in hypoxic chamber with 1% O_2_, 5% CO_2_ and 94% N_2_ for 4 h. After a standard western blot process, the proteins were moved to polyvinylidene fluoride membranes after separation. Subsequently, the membranes were cultivated with primary antibodies against HIF-1α (CST: #36169) and β-Actin (CST: #4967) overnight, followed by incubation with secondary antibodies for visualization by an imaging system. Quantification of the results was conducted using Image-J software.

*Macrophage Polarization and Intracellular Anti-Inflammatory Effect Assessment*: LPS was selected as a stimulus to induce the endogenous oxidative stress against mouse macrophages RAW264.7. RAW264.7 cells were seeded in a 6-well plate at a concentration of 1 × 10^5^ cells per well and incubated for 24 h. Afterward, the cells were treated with 3 μL of 10 ppm LPS together with or without 100 μL of different concentrations of CGO NF with ultrasonic dispersion. Thereby they were divided into four groups: (1) Control, (2) LPS, (3) LPS + 100 ppm CGO NF, (4) LPS + 200 ppm CGO NF and were cocultured for 12 h.

For macrophage polarization assessment, above total macrophages were labeled with PE anti-mouse CD68 and APC anti-mouse CD206 MMR Antibodies (BioLegend). The samples were subsequently analyzed by a FACSCalibur flow cytometry.

For intracellular anti-inflammatory effect assessment by RNA sequencing, total RNA extraction from above macrophages of Control, LPS, and LPS + 100 ppm CGO NF groups and cDNA library preparation were carried out following the manufacturer’s guidelines, and sequencing was performed on an Illumina HiSeq 2500 platform by the Huada Gene Technology Co. Ltd. Three biological replicates were conducted in each group. The R package DESeq2 was employed for analysis of differential expressed genes (DEGs), setting the criteria at |log_2_FoldChange| ≥ 1 and *P* value < 0.05. Kyoto Encyclopedia of Genes and Genomes (KEGG) enrichment analysis of the DEGs was explored via the R package KEGGseq. KEGG terms with corrected *P* values < 0.05 were regarded as significantly enriched in DEGs.

For intracellular anti-inflammatory effect assessment by quantitative real-time polymerase chain reaction (qRT-PCR), gene expression levels of pro-inflammatory factors interleukin-6 (IL-6), tumor necrosis factor (TNF-𝛼), and anti-inflammatory factor IL-10 from above macrophages were measured by qRT-PCR to assess the immunoregulation effect of the CGO NF. Specifically, total RNA was extracted from above macrophages via the total RNA extraction kit (Tiangen Biotech) and complementary DNA (cDNA) was generated using the 5X All-In-One RT Master Mix (G490, Abm). qRT-PCR amplification was conducted with SYBR Green Master Mix (Applied Biosystems) using a QuantStudio 5 Real-Time PCR System (Applied Biosystems) according to the manufacturer’s instructions. Quantitative expression of targeted genes was normalized to housekeeping gene glyceraldehyde-3-phosphate dehydrogenase (GAPDH) and calculated using the 2^-ΔΔCT^ method. The sequences of the primers are listed in Table S1.

For intracellular anti-inflammatory effect assessment by western blots, the expression levels of IL-6, TNF-𝛼 and IL-10 from above macrophages were measured. After a standard western blot process, the proteins were moved to polyvinylidene fluoride membranes after separation. Next, the membranes were cultivated with primary antibodies against IL-6 (CST: #12912), TNF-𝛼 (CST: #11948), IL-10 (CST: #12163) and β-Actin (CST: #4967) overnight, followed by incubation with secondary antibodies for visualization by an imaging system. Quantification of the results was conducted using Image-J software.

*Cell Proliferation Assay*: For cell proliferation assay by 5-ethynyl-2’-deoxyuridine (EdU) incorporation assay, the 3T3 cells (1 × 10^5^ cells/well) were seeded in 6-well plates and incubated for 24 h. These cells were cultured with the CGO NF at different concentrations (0, 1, 2, 3, 4, 5 and 10 ppm, ultrasonic dispersion) for 48 h. EdU assay was carried out utilizing a BeyoClickTM EdU Cell Proliferation Kit with Alexa Fluor 488 (Beyotime Biotech) according to the instructions. The fluorescence microscopy was used to observe the proliferating cells. The cell proliferation ratio was calculated by the formula below:

Cell proliferation ratio (%) = $\frac{Green fluorescent cells}{Blue fluorescent cells}$ × 100% (6)

For cell proliferation assay by CCK-8 assay, after the 3T3 cells were cocultured with the CGO NF at different concentrations (0, 1, 2, 3, 4, 5 and 10 ppm, ultrasonic dispersion) for 24 h, the medium was replaced by CCK-8 reagent. After 2 h of treatment, the absorbance at 450 nm was recorded by a microplate reader.

*Cell Migration Assay*: After culturing 3T3 cells, scratches were created with a 200 μL pipette tip. After that, 3 ppm of CGO NF in DMEM (ultrasonic dispersion) was cocultured, and the control group was treated with normal medium only. The recovery area of the scratch was observed under the fluorescence microscopy at 0, 5, 10 and 20 h, respectively. Image-J software was utilized to quantify the area of the initial scratch (*S_0_*) and healing scratch (*S_t_*). Area recovery ratio was calculated using the following formula:

Area recovery ratio (%) = $\frac{S_{0}-S_{t}}{S_{0}}$ × 100% (7)

*Wound Infection Therapy and Healing*: All animal experiments comply with the Principles of Laboratory Animal Care (People’s Republic of China) and the Institutional Animal Use and Care Committee of Xinxiang Medical University (No. XYLL-20240274). 6-week-old female Balb/c mice (15-20 g) were purchased from the Experimental Animal Center of Beijing Wei Tong Li Hua Experimental Animal Technology Co., Ltd. The effectiveness of wound infection therapy and healing was evaluated by constructing a full-thickness wound model. Briefly, pentobarbital sodium (1%) was intraperitoneally injected to anesthetize mice according to a dose of 40 mg/kg. Next, a round full-thickness skin wound with a diameter of 8 mm was created on the shaved back using a hole punch under sterile conditions. Finally, 100 μL of prepared 10^7^ CFU/mL *S. aureus* suspension was dropped into the round wound and all mice were infected for 2 days.

*S. aureus* infected wound mice were randomized into six groups (*n* = 6) as follows: (1) PBS (100 μL), (2) H_2_O_2_ (100 μL, 10 mM), (3) PBS + NIR, (4) H_2_O_2_ + NIR, (5) CGO NF (a 6-mm-diameter round NF) + H_2_O_2_, and (6) CGO NF + H_2_O_2_ + NIR. NIR laser irradiation (808 nm, 0.3 W/cm^2^) was performed on the PBS + NIR group, H_2_O_2_ + NIR group, and CGO NF + H_2_O_2_ + NIR group after 0.5 h of dosing treatment at the infection site for 10 min. The thermal images of the mice and temperature change at the infection sites were recorded on the thermal imaging camera. The wounds were photographed and the weights were measured every other day for 10 days. It is noted that the CGO NF as a wound dressing was replaced every day. The wound area was determined by Image-J software. On the 5th day, wound tissues were harvested to make paraffin sections for dihydroethidium (DHE) immunofluorescence staining. On the 10th day, bacteria in the skin wound were collected and quantified using SPM. Meanwhile, wound tissues were harvested to make paraffin sections for histological (hematoxylin and eosin (H&E), Giemsa, Masson) and immunofluorescence (TNF-𝛼, vascular endothelial growth factor (VEGF)) staining. The major organs (heart, liver, spleen, lung, and kidney) of mice from the therapy group were dissected to make paraffin sections and stained with H&E. The histological staining was observed by the fluorescence microscopy and the immunofluorescence staining was recorded by the CLSM.

*In Vivo Biosafety and Biodistribution*: The healthy mice were anesthetized intraperitoneally with 1% pentobarbital sodium (40 mg/kg) in the beginning, and the skin on anterior upper backs (the subcutaneous surgical site) was shaved and disinfected. Afterward, the skin was sheared layer by layer under sterile conditions, and ready round CGO NFs (6 mm in diameter) were subcutaneously intercalated (one NF was intercalated into each mouse). Subsequently, the incisions were sutured. After that, the body weights of mice were measured. At the 1st, 3rd, 7th, 15th and 30th day (six mice per time point) post intercalation, such CGO intercalated mice were euthanized. Another six healthy mice were used as the control. Before the mice were euthanatized, blood samples (~1 mL) were collected for blood biochemistry and complete blood panel analyses. Major organs of those mice were dissected and divided into two halves for histological examination and biodistribution measurement, respectively. For histological examination, the major organs including heart, liver, spleen, lung and kidney were dissected from each mouse, to make paraffin sections for further H&E staining, and then examined by the fluorescence microscopy. For biodistribution measurement, major organs including heart, liver, spleen, lung, kidney, stomach, intestine, skin, muscle and bone from the mice were solubilized by aqua regia for ICP-AES measurement to determine the Cu and Ge contents in these different organs.

*Statistical Analysis*: Quantitative data were presented as mean ± SD for independent experiments with a sample size *n* ≥ 3. *P* values were estimated by one-way ANOVA statistical analysis method to evaluate significance among experimental data. *P* value of 0.05 was considered significant. The results are labelled in Figures as **P* < 0.05, ***P* < 0.01, and ****P* < 0.001. n.s indicates not significant difference between compared two groups.

**Supplementary Tables and Figures**

**Table S1. Summary of solution-state concentration ranges, and film-state test conditions of the CGO NF adopted for performance evaluation in this study.**

| Experimental Data | Status of CGO NF |
| --- | --- |
| Catalytic Activity (POD, CAT, and GSH depletion) | Solution, 100 ppm |
| In vitro antibacteria/antibiofilm | Film, a 6-mm-diameter round |
| In vitro ROS tests | Film, a 6-mm-diameter round |
| Intracellular O_2_ Generation ([Ru(dpp)_3_]Cl_2_) | Solution, 0, 50 and 100 ppm |
| HIF western blot | Solution, 0, 25, 50 and 100 ppm |
| Flow cytometry of macrophage polarization | Solution, 100 and 200 ppm |
| RNA sequencing | Solution, 100 ppm |
| qRT-PCR | Solution, 100 ppm |
| Anti-inflammatory western blot | Solution, 100 and 200 ppm |
| EdU  Cell proliferation assay by CCK-8  Cell migration assay  In vivo wound therapy | Solution, 0, 1, 2, 3, 4, 5 and 10 ppm  Solution, 0, 1, 2, 3, 4, 5 and 10 ppm  Solution, 3 ppm  Film, a 6-mm-diameter round |

**Table S2.** Primers used in the present study for qRT-PCR.

| Target Direction Primer sequence (5’→3’)  gene |
| --- |
| m-IL-6 F ACAAAGCCAGAGTCCTTCAGAG  m-IL-6 R TGTGACTCCAGCTTATCTCTTGG  m-TNF-α F TATGGCCCAGACCCTCACA  m-TNF-α R GGAGTAGACAAGGTACAACCCATC  m-IL-10 F GGCGCTGTCATCGATTTCTC  m-IL-10 R ATGGCCTTGTAGACACCTTGG  m-GAPDH F AGGTCGGTGTGAACGGATTTG  m-GAPDH R TGTAGACCATGTAGTTGAGGTCA |


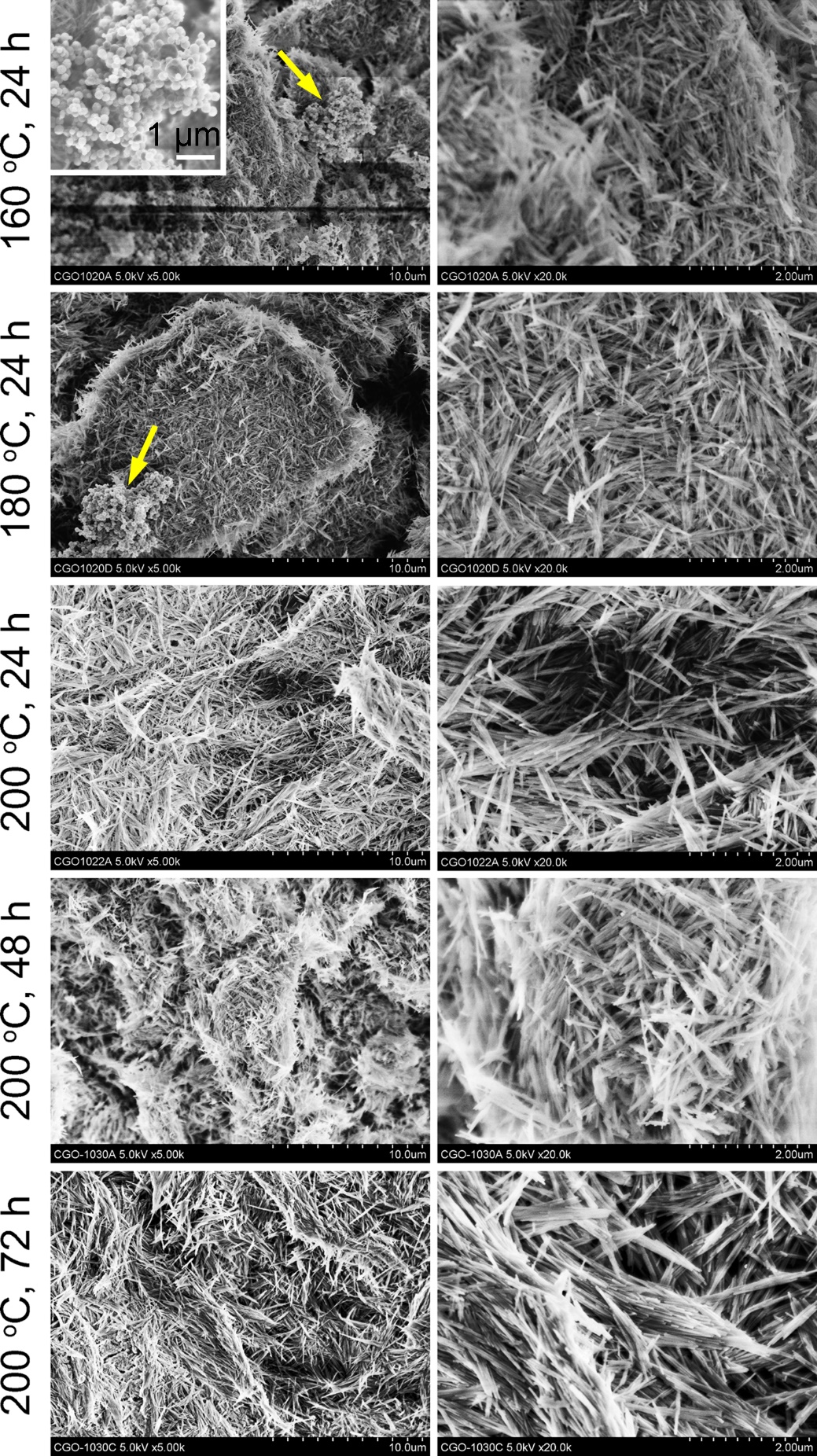


**Figure S1.** SEM images of the CGO NF synthesized at various temperature for varying time in different magnifications. Yellow arrows indicate the agglomerations consisting of many small nanospheres.

Previous studies have revealed that assembling nanowires into macroscopic films via filtration strategy requires nanofibrous materials possessing higher aspect ratio [5]. To obtain optimized CGO NF, it is worthwhile to develop a controllable hydrothermal method to synthesize CGO nanowires. First, to explore the impact of temperature on the shape evolution, the reaction temperature changed from 180 to 200 °C while the reaction time was 24 h. The SEM images (Figure S1) exhibit that when the temperature was controlled at 160 and 180 ^o^C, there exist agglomerations consisting of many small nanospheres besides nanowires, possibly attributed to incompletely transformed Cu(OH)_2_ or/and GeO_2_ [6]. When the reaction temperature reached 200 ^o^C, the nanowires are monodisperse (Figure S1). The results illustrate that the reaction temperature plays a vital role in the formation of unform nanowires. Hence, the reaction temperature was fixed at 200 °C in further investigation.


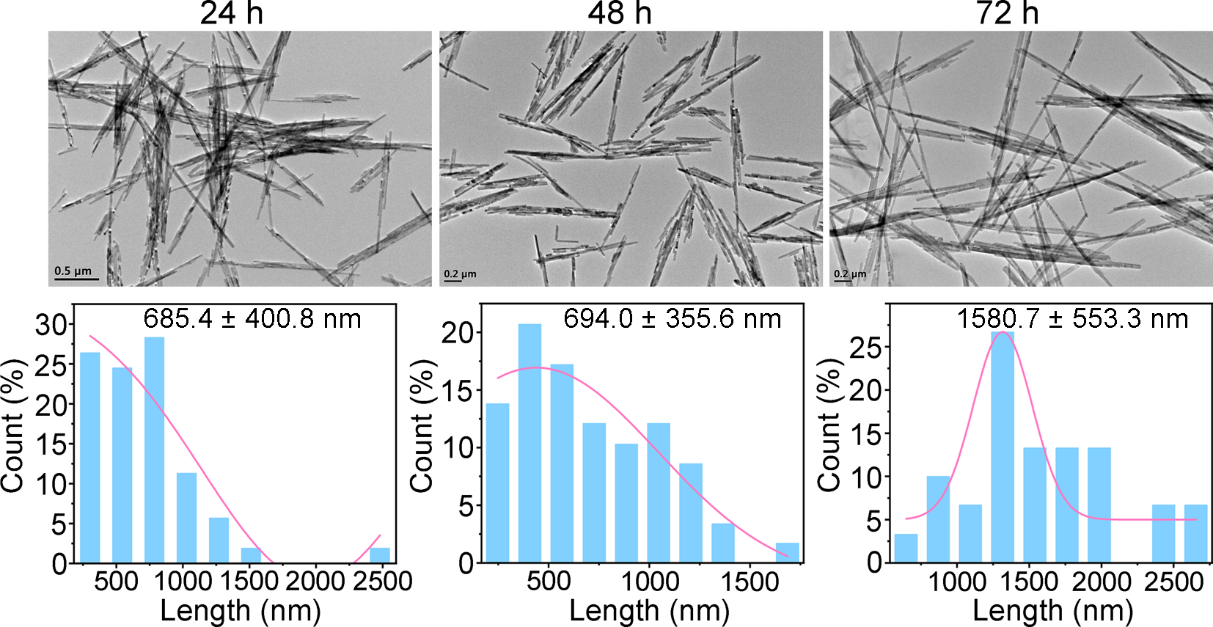


**Figure S2.** TEM images of the CGO NF synthesized at 200 ^o^C for different time and corresponding statistical analysis of length distribution from TEM.

Furthermore, the influence of the reaction time on the length of the nanowires was studied. The TEM images in Figure S2,S3 show that the nanowires with different length were achieved after maintaining at 200 ^o^C for different time, demonstrating the reaction time plays a crucial role in control of the nanowire length. In detail, the length grew continuously from 685.4 nm to 694.0 nm, 1580.7 nm, and 2.3 μm when the reaction time was 24, 48, 72 and 96 h, respectively (Figure S2-S4). In this work, considering the longer nanowire, as stated above, giving rise to better effect of film formation, and the cost-effective synthesis, finally we choose 200 ^o^C and 96 h as the reaction temperature and time, respectively, to obtain the optimized CGO NF.


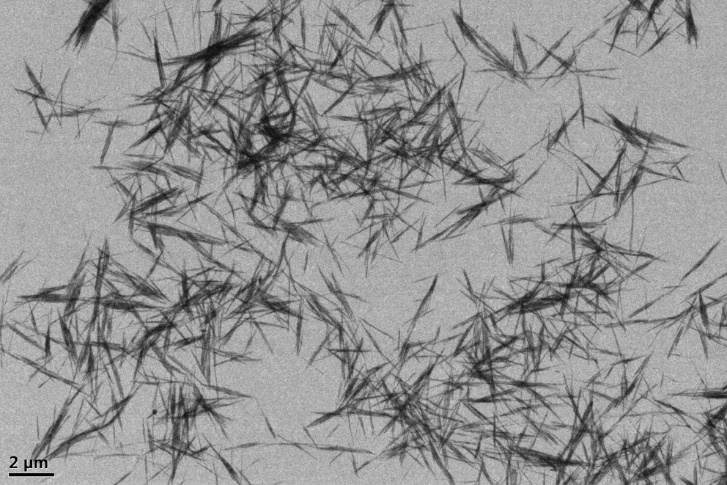


**Figure S3.** TEM image of the CGO NF synthesized at 200 ^o^C for 96 h.


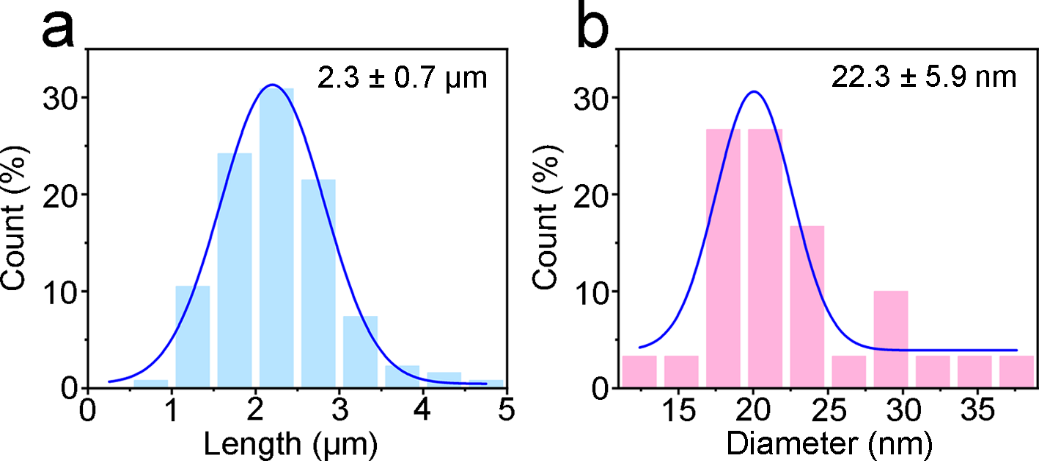


**Figure S4.** Statistical analysis of (a) length and (b) diameter distribution of the CGO NF synthesized at 200 ^o^C for 96 h based on the TEM observation.


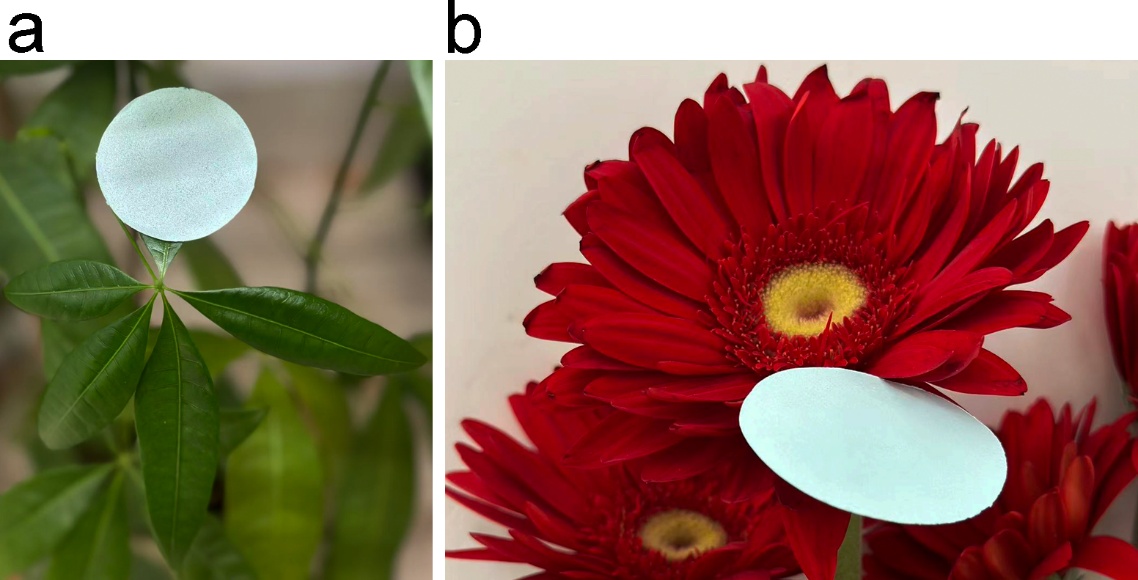


**Figure S5.** Photo showing the lightweight characteristic of the obtained CGO NF. The entire film could be stably supported on (a) a leaf or (b) the petals of a flower, highlighting its lightweight characteristic.


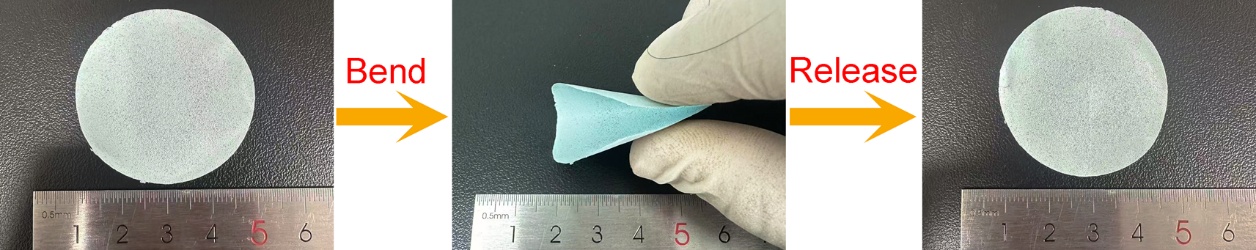


**Figure S6.** Photos for dynamical bending process of the CGO NF.


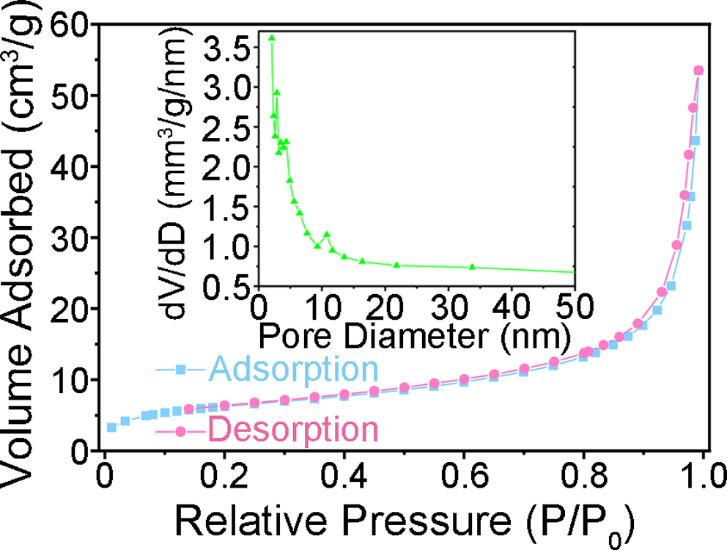


**Figure S7.** Nitrogen adsorption-desorption isotherms of the CGO NF. Inset is the pore size distribution.


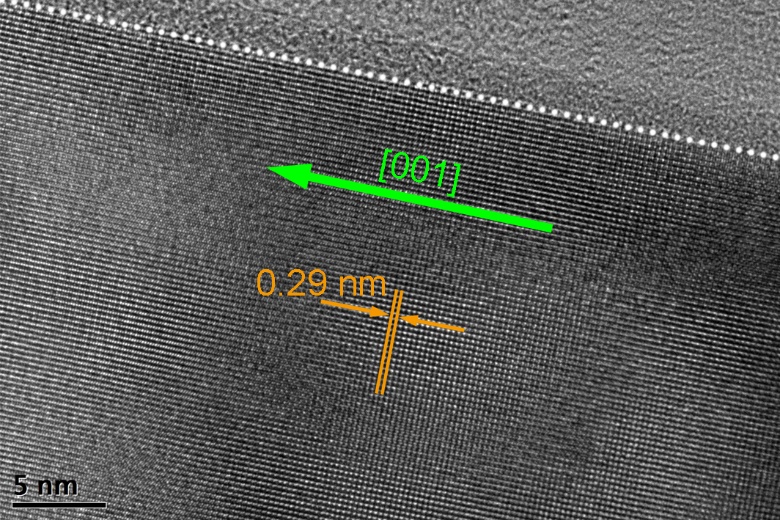


**Figure S8.** HRTEM of an individual CGO nanowire, indicating the nanowire grows along the [001] direction.


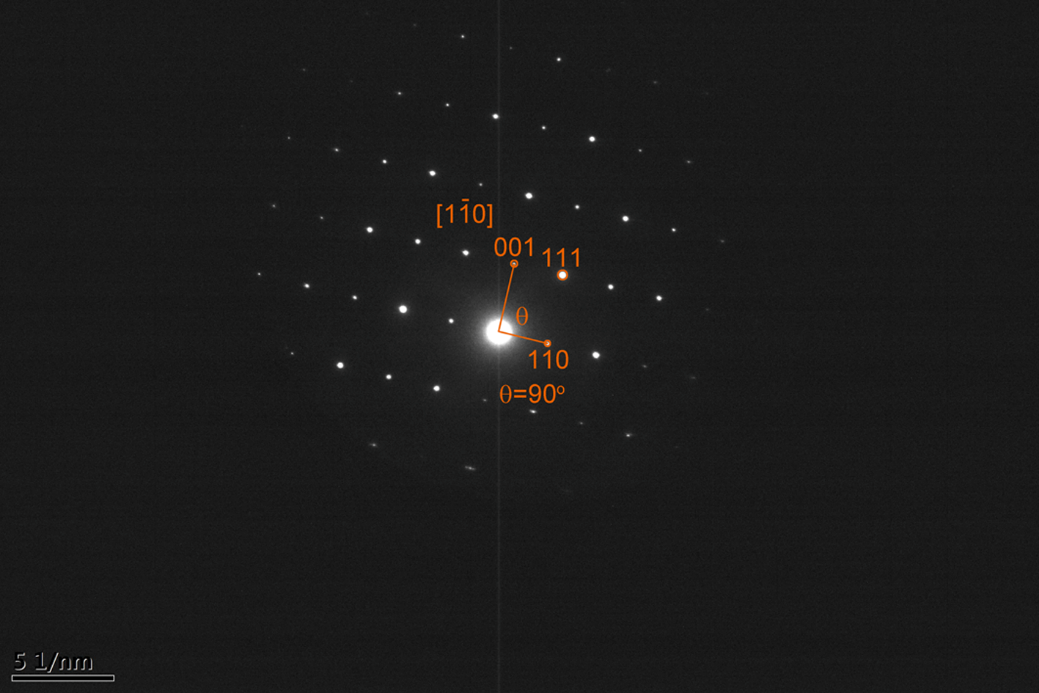


**Figure S9.** SAED pattern of an individual CGO nanowire.


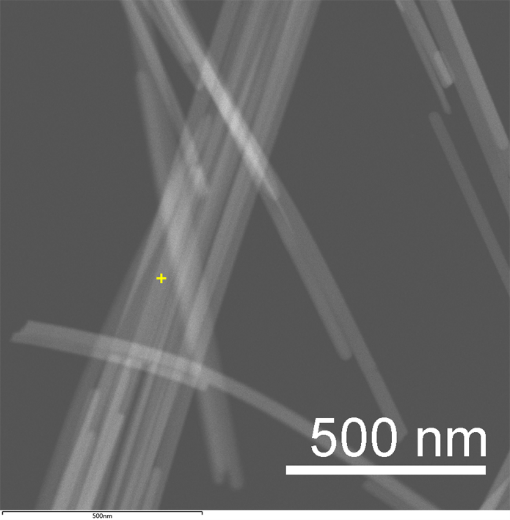


**Figure S10.** STEM image of the CGO NF.


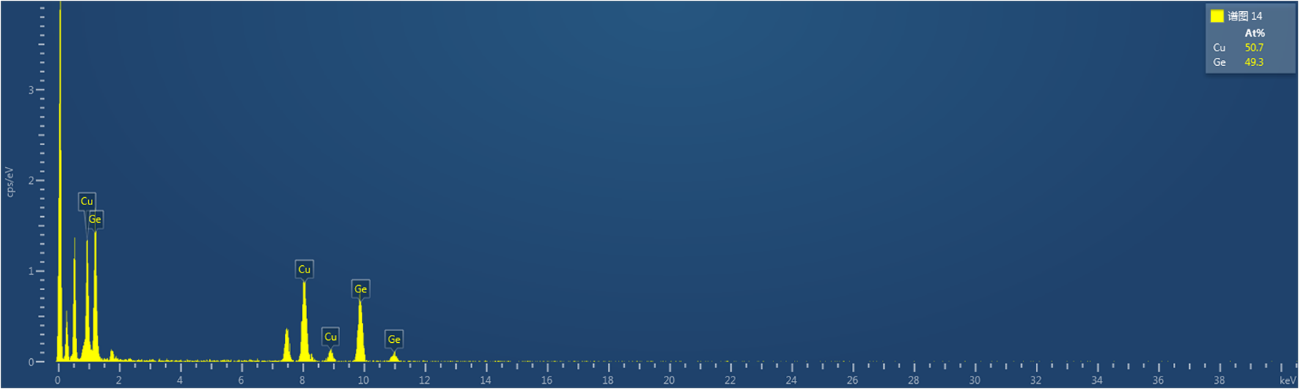


**Figure S11.** EDS spectrum taken from a spot on an individual CGO nanowire (indicated by a yellow plus sign in Figure S10).


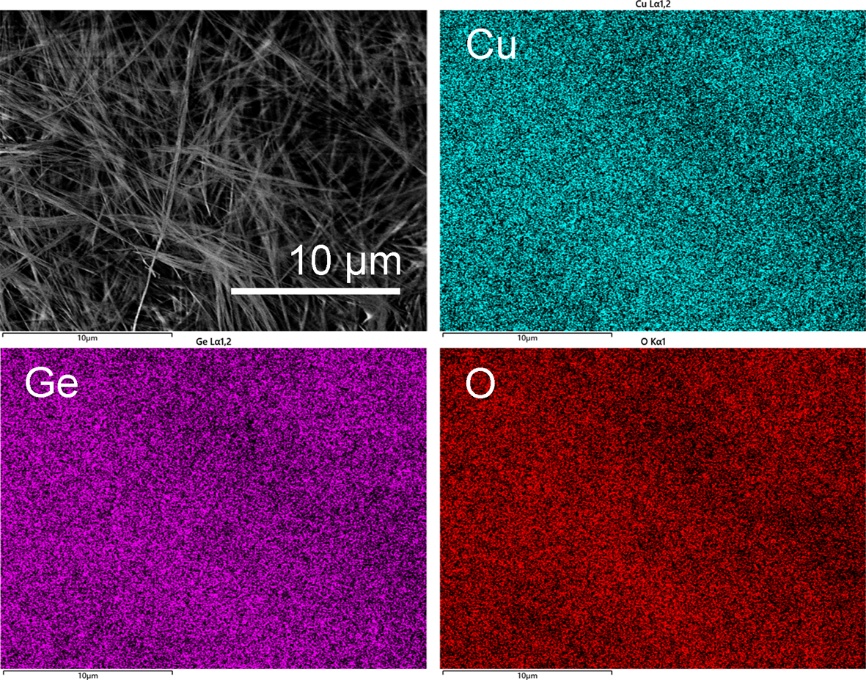


**Figure S12.** SEM-EDS elemental mapping images of the CGO NF.


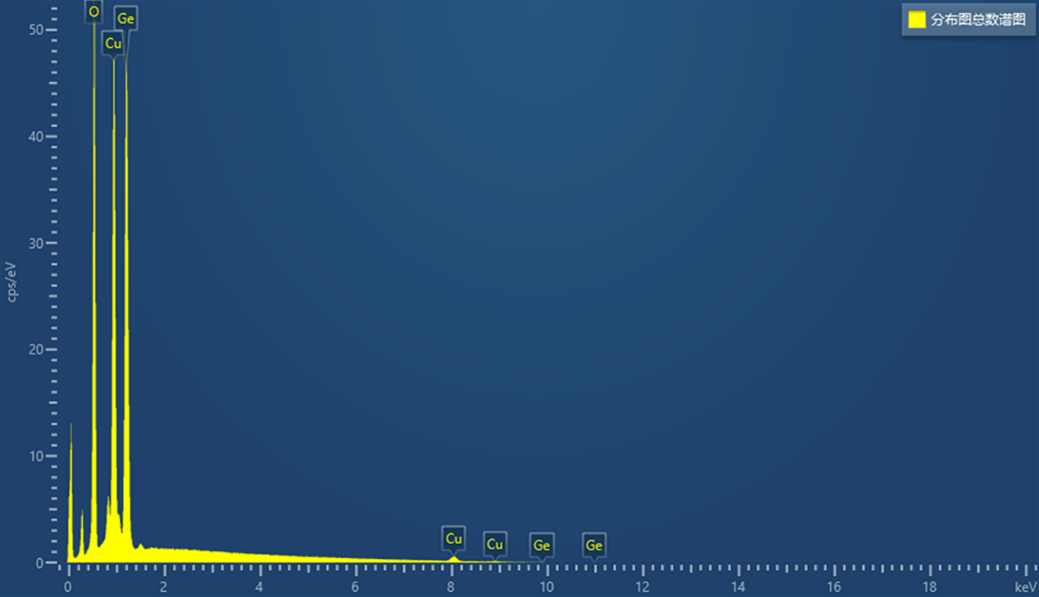


**Figure S13.** EDS spectrum of the CGO NF acquired from the region of the SEM image (Figure S12).


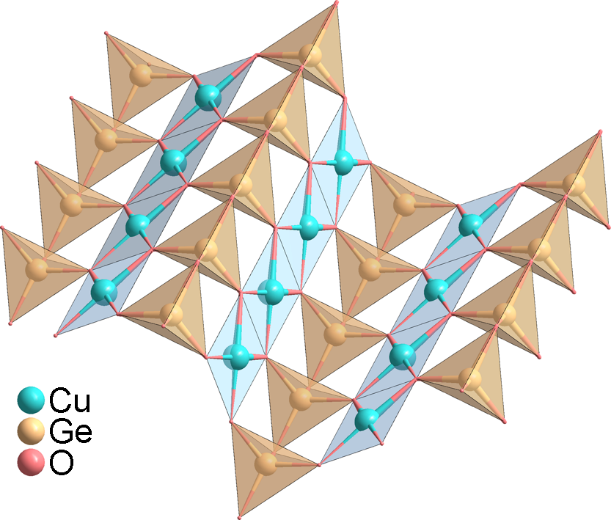


**Figure S14.** Crystal structure of CGO formed by edge-sharing CuO_4_ squares and corner-sharing GeO_4_ tetrahedra.


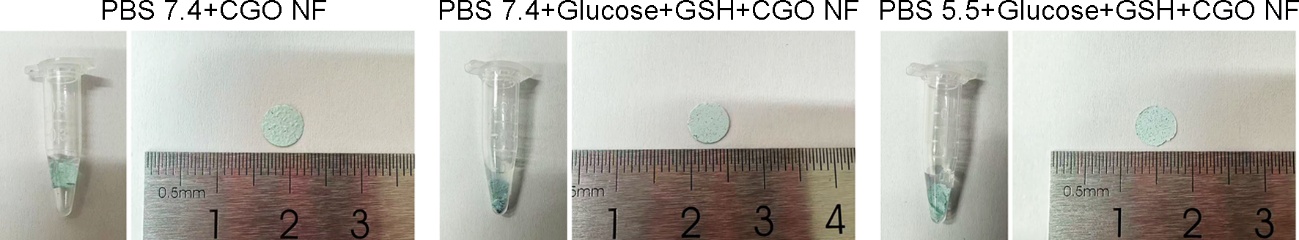


**Figure S15.** Photos showing the CGO NFs and the CGO NFs placed in PBS (pH 7.4), PBS (pH 7.4) + Glucose (25 mM) + GSH (8 mM), and PBS (pH 5.5) + Glucose (25 mM) + GSH (8 mM) for evaluating their photothermal performance.


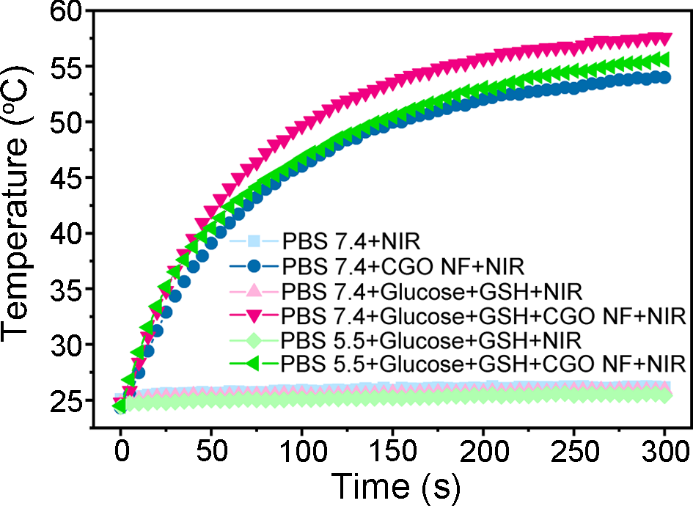


**Figure S16.** Temperature elevation curves of PBS (pH 7.4), PBS (pH 7.4) + CGO NF, PBS (pH 7.4) + Glucose (25 mM) + GSH (8 mM), PBS (pH 7.4) + Glucose (25 mM) + GSH (8 mM) + CGO NF, PBS (pH 5.5) + Glucose (25 mM) + GSH (8 mM), and PBS (pH 5.5) + Glucose (25 mM) + GSH (8 mM) + CGO NF under 808 nm NIR laser irradiation of 0.7 W/cm^2^.


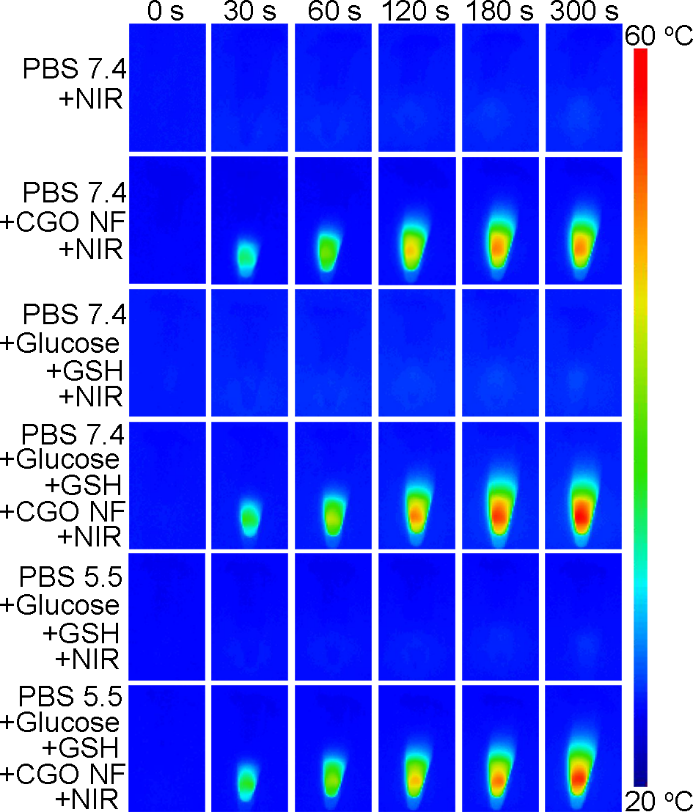


**Figure S17.** IR thermal imaging of PBS (pH 7.4), PBS (pH 7.4) + CGO NF, PBS (pH 7.4) + Glucose (25 mM) + GSH (8 mM), PBS (pH 7.4) + Glucose (25 mM) + GSH (8 mM) + CGO NF, PBS (pH 5.5) + Glucose (25 mM) + GSH (8 mM), and PBS (pH 5.5) + Glucose (25 mM) + GSH (8 mM) + CGO NF under 808 nm NIR laser irradiation of 0.7 W/cm^2^.


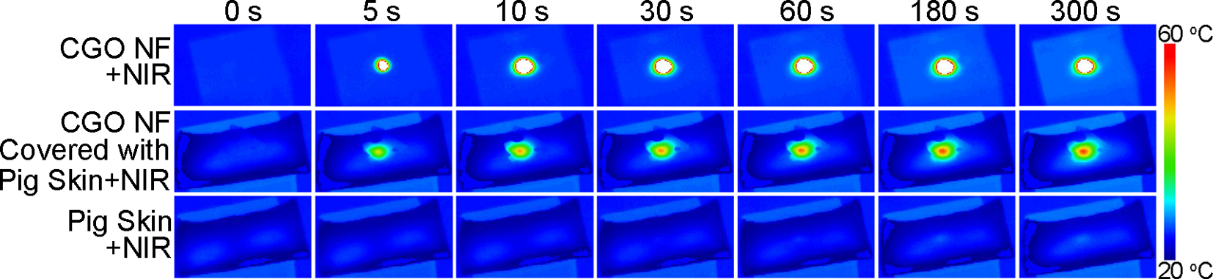


**Figure S18.** IR thermal imaging of a CGO NF covered with a pig skin, the CGO NF, and the pig skin under 808 nm laser irradiation of 1.0 W/cm^2^.


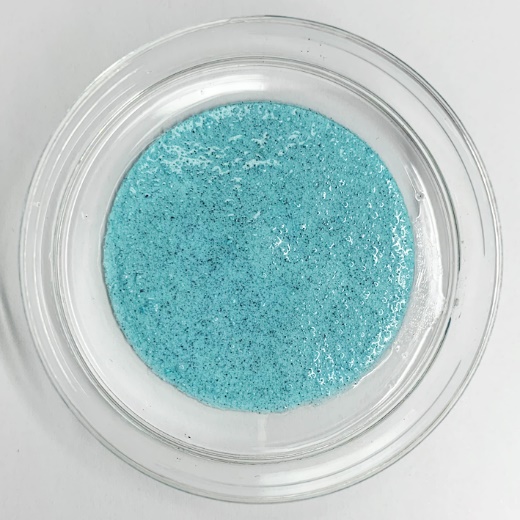


**Figure S19.** Photo of a CGO NF immersed in H_2_O.


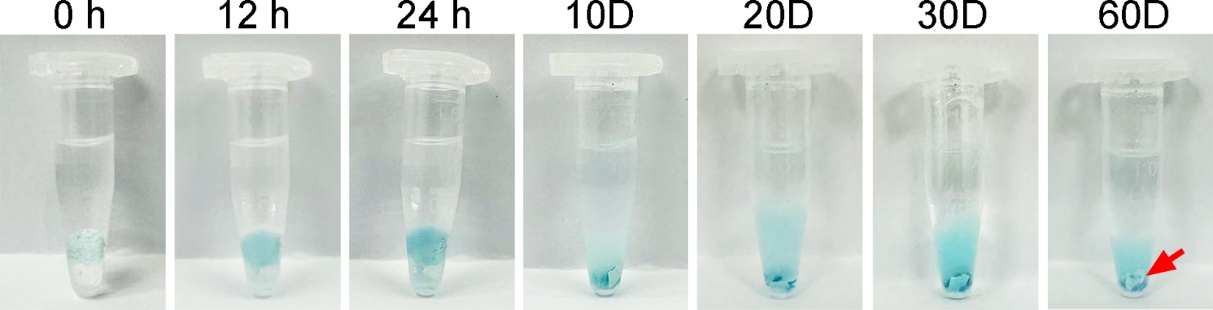


**Figure S20.** Photos of the CGO NF immersed in the solution (without ultrasonic dispersion) in the presence of 25 mM glucose and 8 mM GSH in PBS (pH 5.5) before and after varying time points.


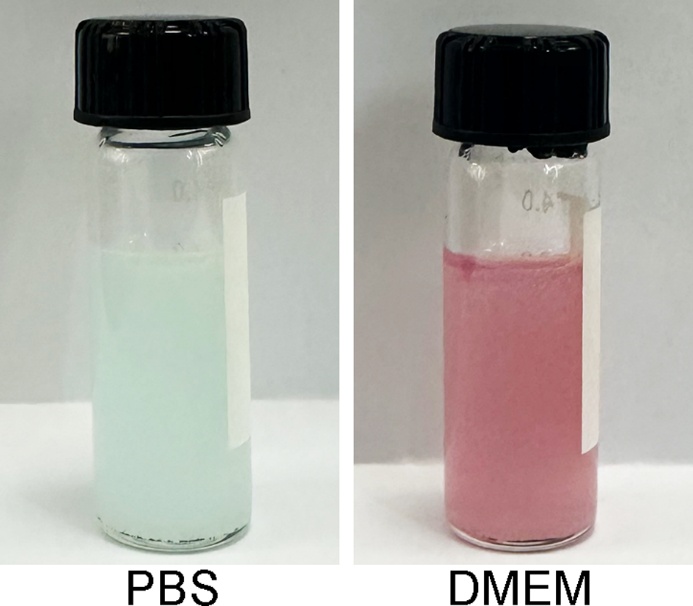


**Figure S21. Photos of the CGO NF dispersed in PBS and DMEM (2000 ppm) using sonication.**


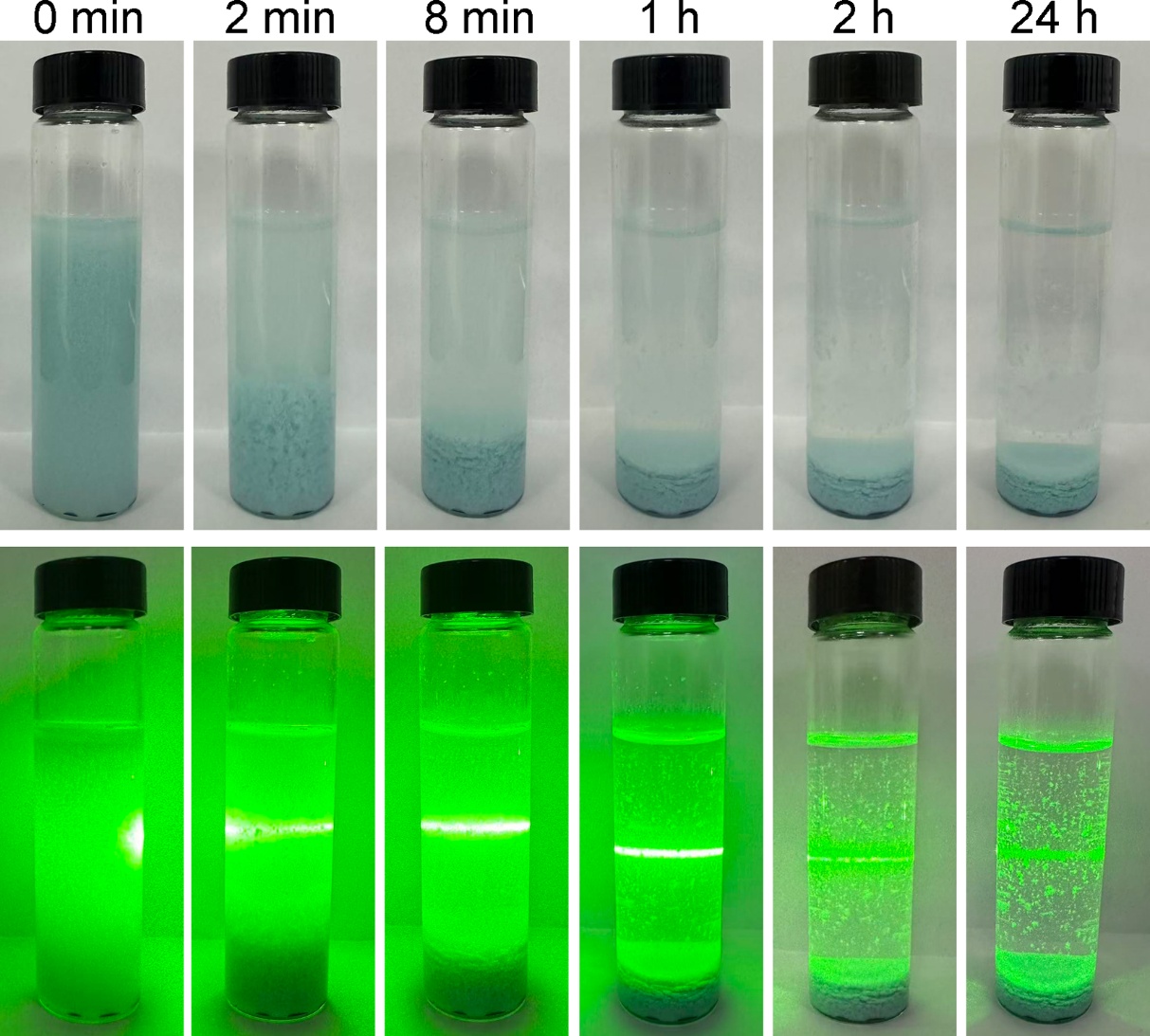


**Figure S22. Photos of the CGO NF ultrasonically dispersed in water exhibiting Tyndall effect before and after different time points.**


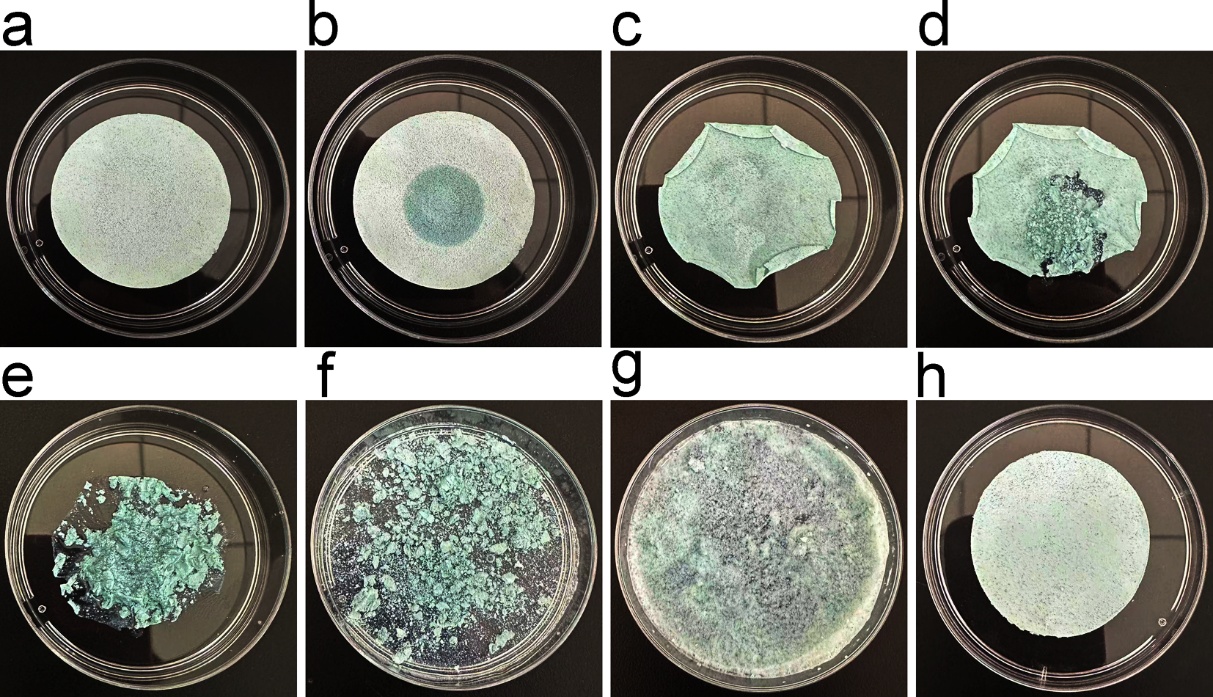


**Figure S23. Photos of the CGO NF (a) before and (b–g) after adding gradually increased amounts of water onto its surface. (h) Photo of the CGO NF after redispersion in deionized water and vacuum filtration.**


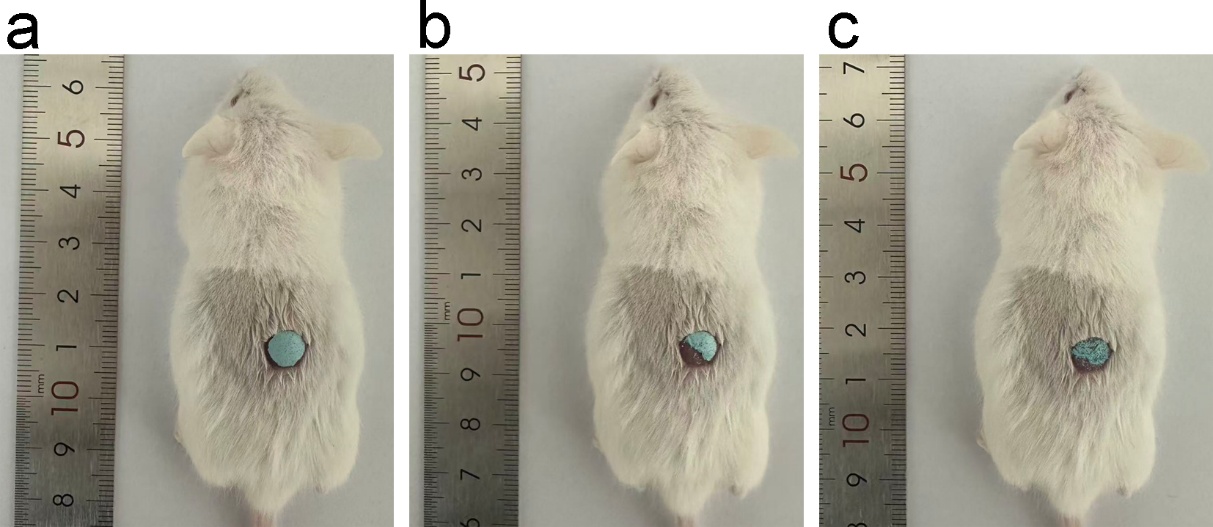


**Figure S24. Photos of the CGO NF on the wound (a) before and (b,c) after adding gradually increased amounts of PBS onto its surface.**


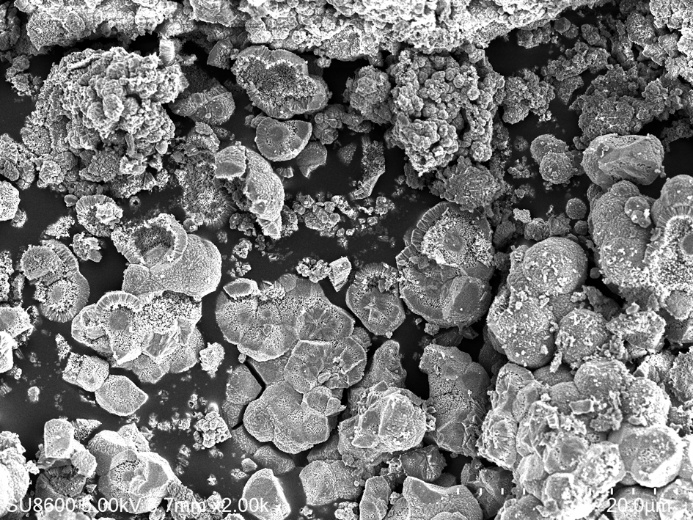


**Figure S25.** SEM image of bulk GeO_2_.


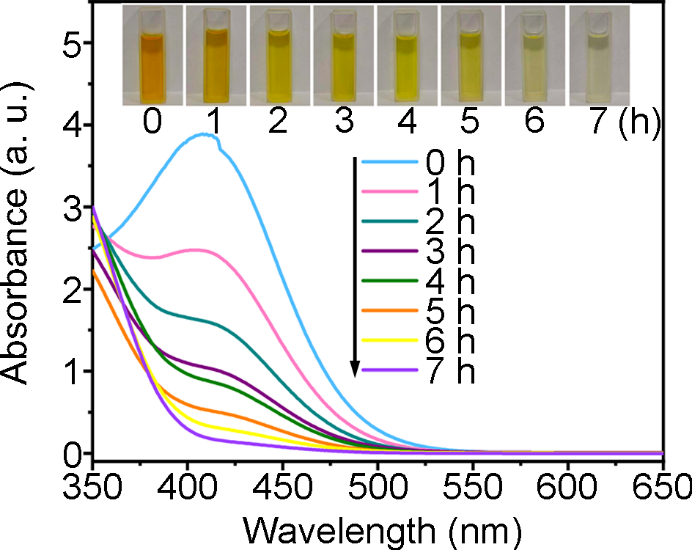


**Figure S26.** UV-Vis absorption spectra and corresponding color changes of GSH incubated with DTNB in PBS (pH 7.4) treated with bulk GeO_2_ for different time points.


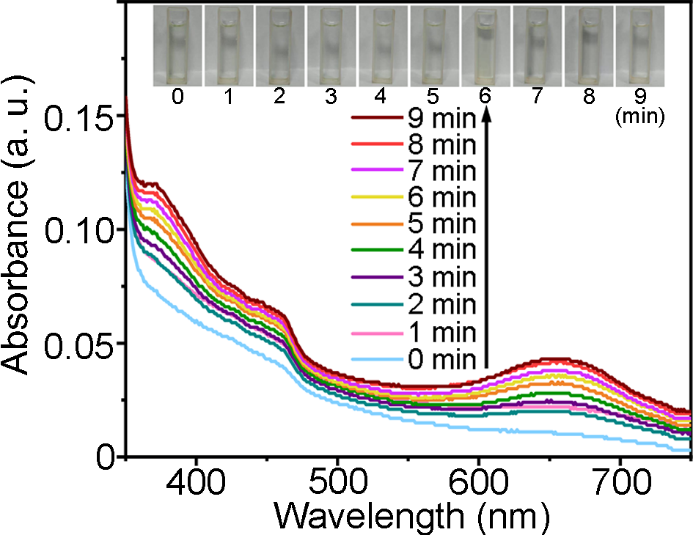


**Figure S27.** Time-dependent absorption changes of TMB and corresponding color changes in the presence of bulk GeO_2_ and H_2_O_2_ in PBS (pH 5.5).


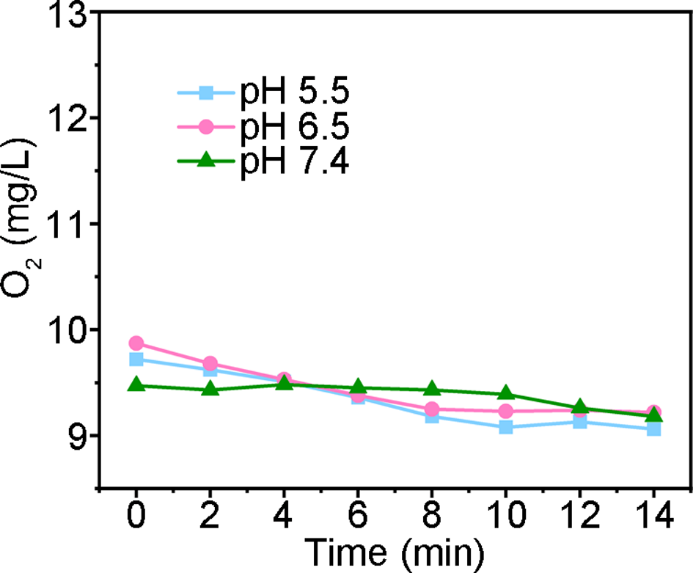


**Figure S28.** O_2_ generation from solutions in the presence of bulk GeO_2_ and H_2_O_2_ in PBS with varied pH.


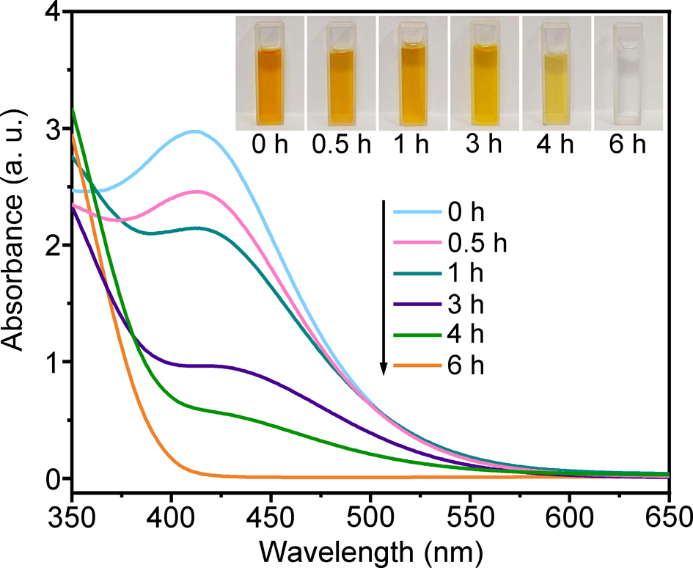


**Figure S29.** UV-Vis absorption spectra and corresponding color changes of GSH incubated with DTNB in PBS (pH 7.4) containing 25 mM glucose treated with CGO NF for different time points.


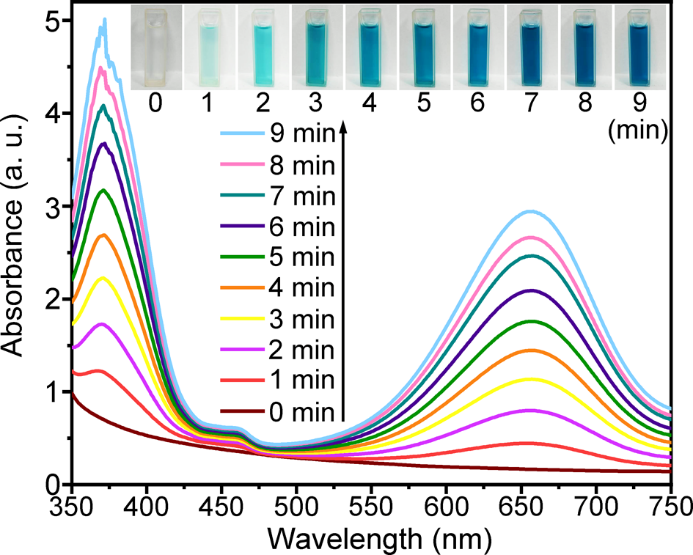


**Figure S30.** Time-dependent absorption changes of TMB and corresponding color changes in the presence of the CGO NF, H_2_O_2_, 25 mM glucose and 8 mM GSH in PBS (pH 5.5).


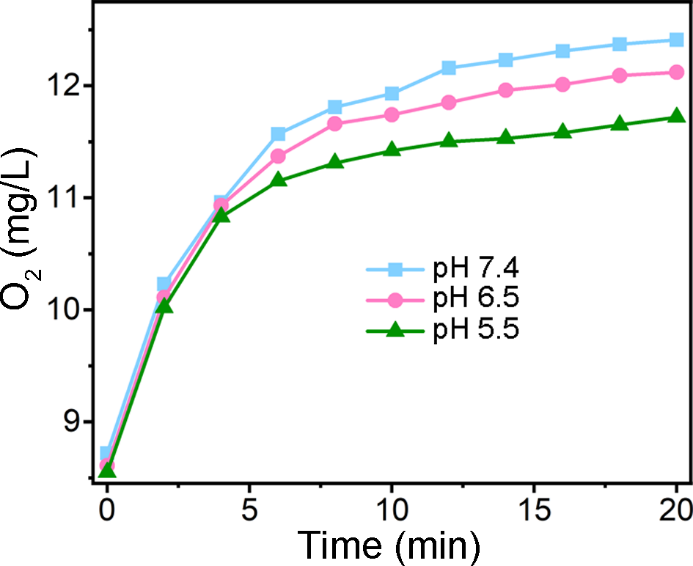


**Figure S31.** O_2_ generation from solutions in the presence of the CGO NF, H_2_O_2_ and 25 mM glucose in PBS with varied pH.


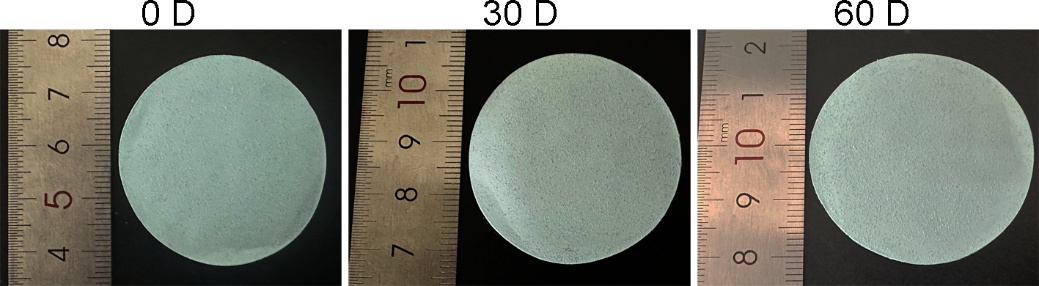


**Figure S32.** Photos of the CGO NF before and after being stored at room temperature in air for different time points.


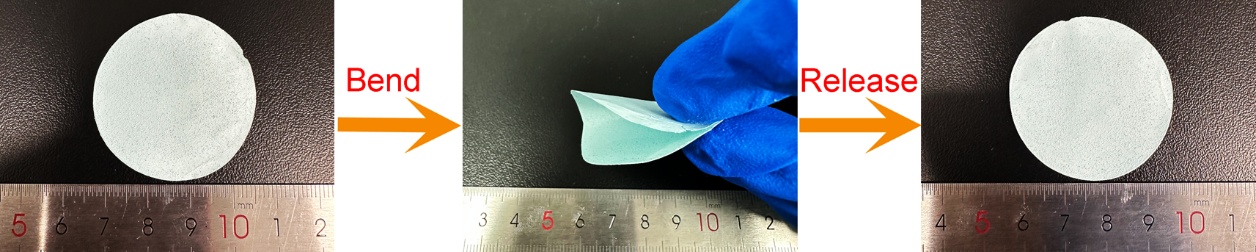


**Figure S33.** Photos for dynamical bending process of the CGO NF after being stored at room temperature in air for 60 d.


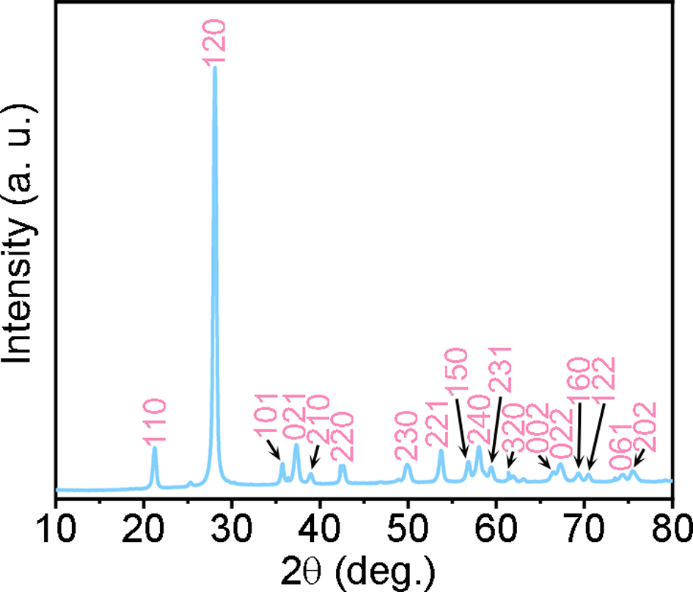


**Figure S34.** XRD pattern of the CGO NF after being stored at room temperature in air for 60 d.

To investigate the stability of the CGO NF exposed to air, the CGO NF was stored at room temperature in air for 60 d. As can be seen from **Figure S32**, no differences were observed in the CGO NF before and after storage. Notably, the flexibility of the CGO NF was well maintained after 60 d of storage (**Figure S33**). Furthermore, XRD was conducted to confirm the change of crystalline structure of the CGO NF after being stored at room temperature in air for 60 d (**Figure S34**). The XRD pattern shows the sample is indexed to the orthorhombic CGO (JCPDS 74-0302). No characteristic peaks from other impurities are found in the XRD pattern. These findings suggest the CGO NF remained stable after storage at room temperature in air for 60 d.


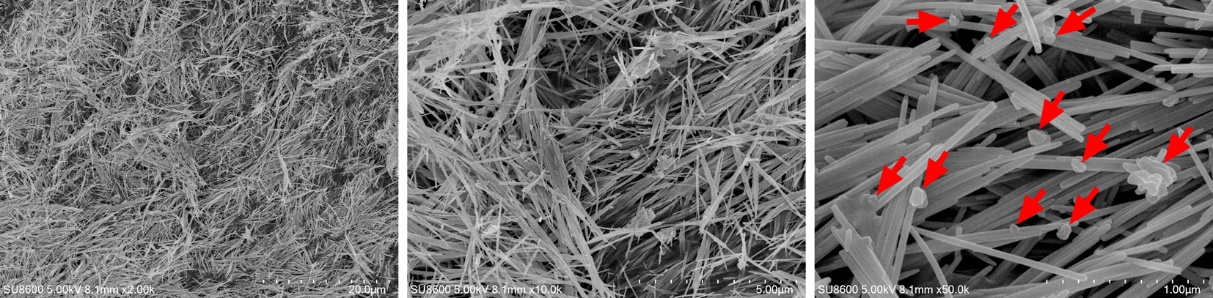


**Figure S35.** SEM images of the CGO NF immersed in the solution (without ultrasonic dispersion) in the presence of 25 mM glucose and 8 mM GSH in PBS (pH 5.5) for 60 d in different magnifications.

Next, we explored the stability of the CGO NF under diabetic-mimicking conditions by immersing it into PBS (pH 5.5) containing glucose and GSH (without ultrasonic dispersion). The results reveal that the CGO NF gradually dispersed in the solution, but rapid degradation did not occur. As indicated by the red arrows, the blue fragments remained even after 60 d of storage (**Figure S20**). SEM images of such residual fragments are shown in **Figure S35**. As seen, these fragments are still composed of intact and well-defined nanowires, with only a few particles from partial degradation (indicated by the red arrows). These results demonstrate that the CGO NF remains relatively stable in simulated diabetic wound environments.

**
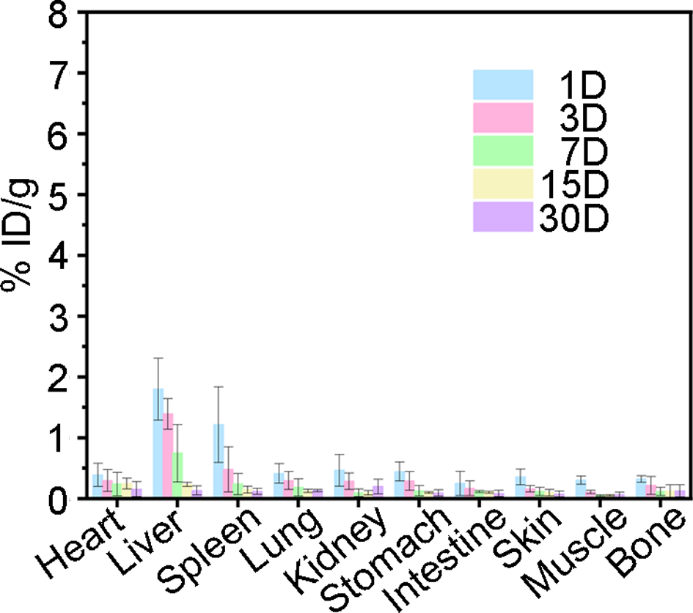
**

**Figure S36.** Biodistribution of Ge in major organs at different time points post intercalation of the CGO NFs.


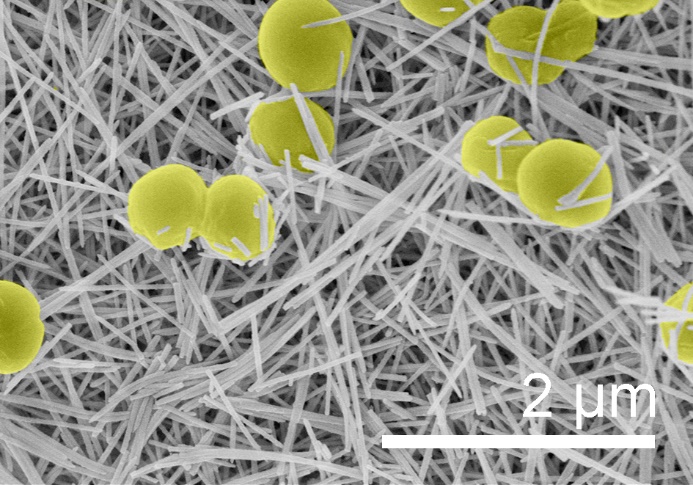


**Figure S37.** SEM image of the CGO NF adsorbing *S. aureus* bacteria. Yellow spheres indicate *S. aureus*.


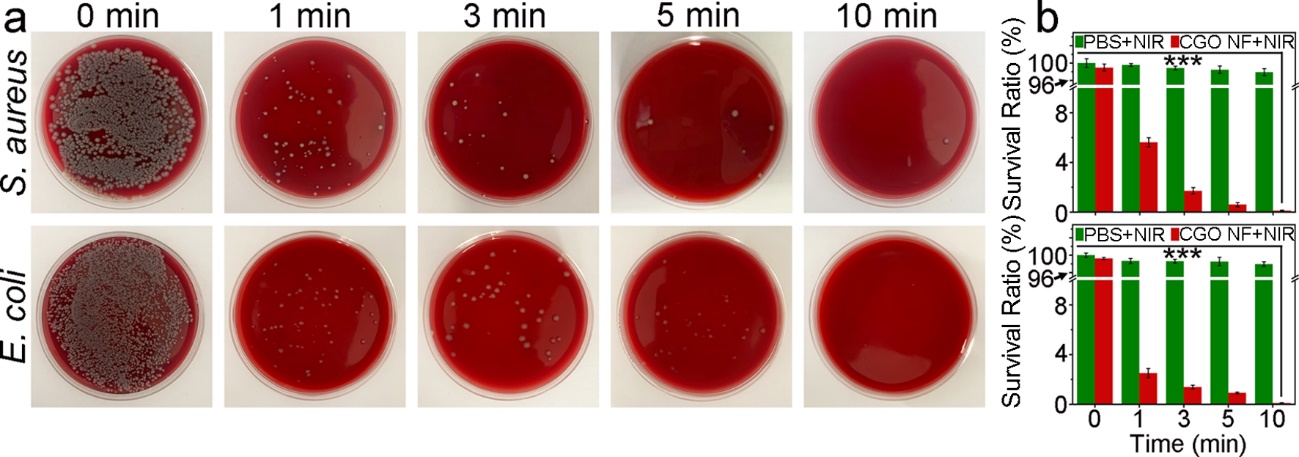


**Figure S38.** (a) Photos and (b) quantitative analysis of bacterial colonies formed by *S. aureus* and *E. coli* planktonic bacteria treated with the CGO NF after 808 nm laser irradiation (0.5 W/cm^2^) for different time.


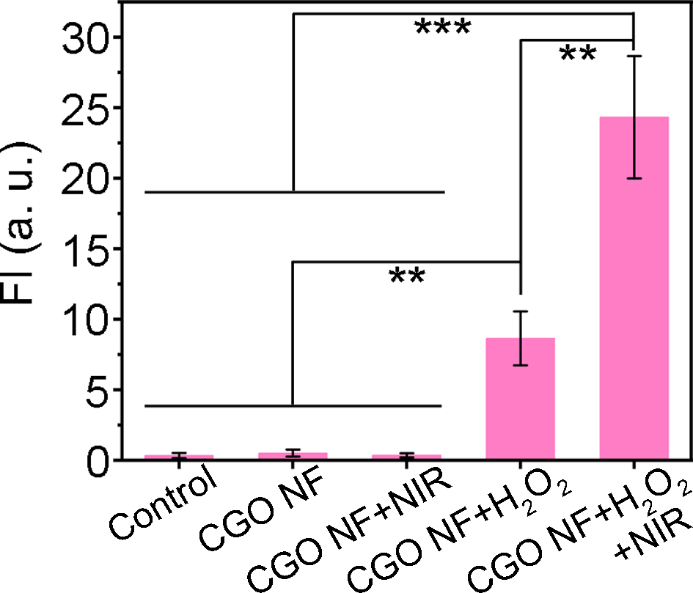


**Figure S39.** Fluorescence intensity of ROS in *S. aureus* bacteria after different treatments.


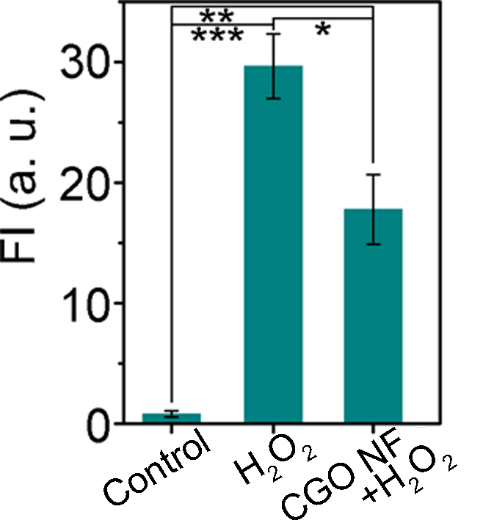


**Figure S40.** Fluorescence intensity of ROS in 3T3 cells after various treatments.


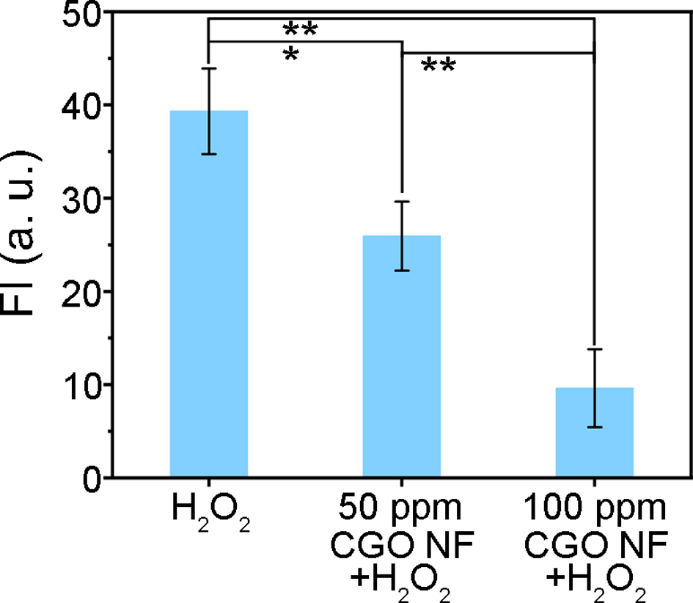


**Figure S41.** Fluorescence intensity of O_2_ probe [Ru(dpp)_3_]Cl_2_ in 3T3 cells after different treatments.


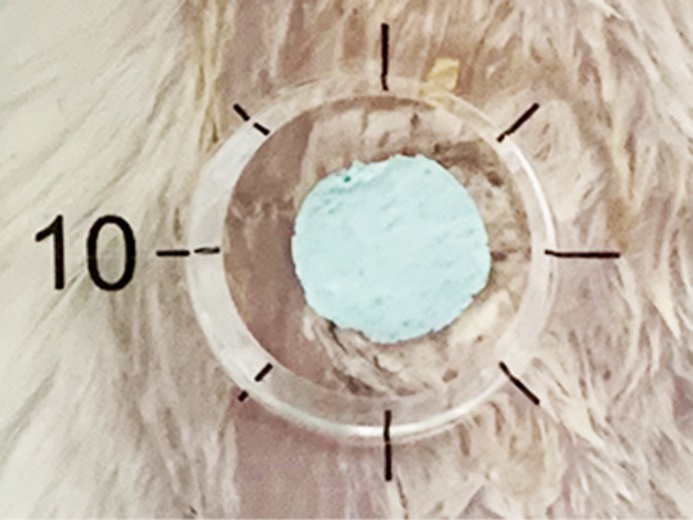


**Figure S42.** Photo of the CGO NF on a wound infection model as a wound dressing for wound infection therapy and healing.


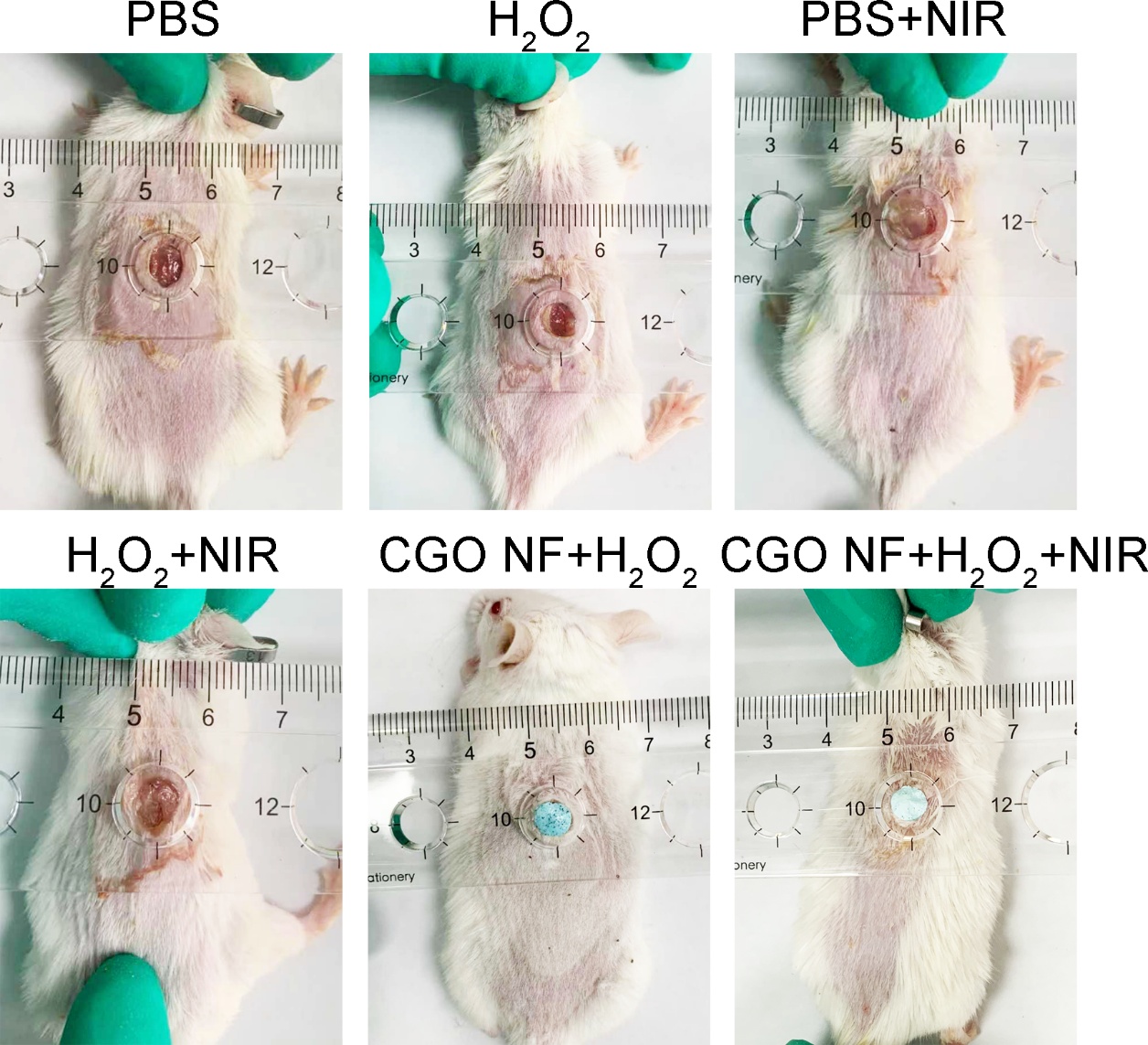


**Figure S43.** Representative photos of infected wounds at day 0.


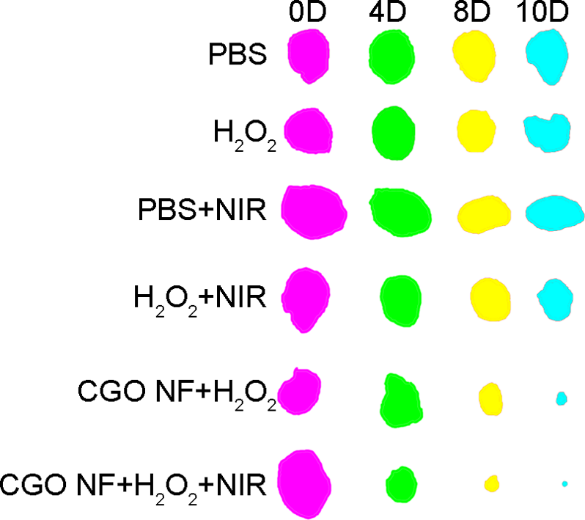


**Figure S44.** Schematic diagram of time-evolved wound areas after different treatments.


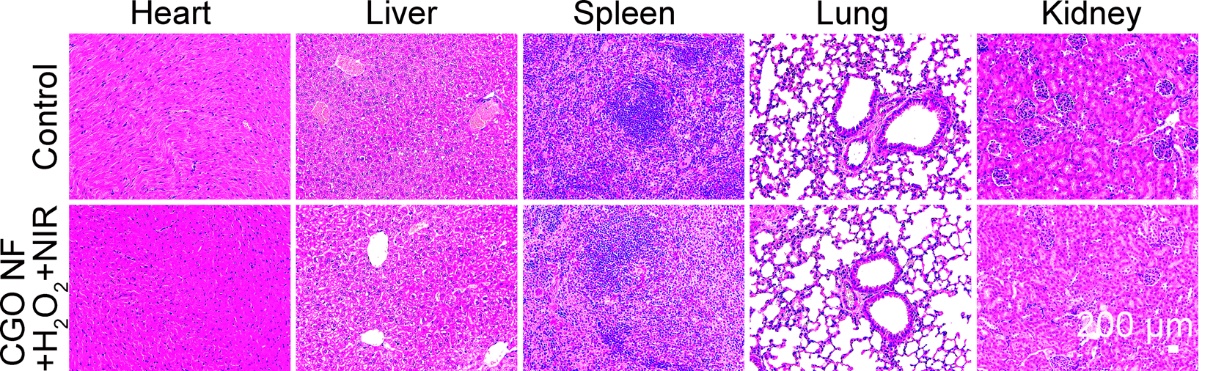


**Figure S45.** H&E-stained histological images of major organs 10 days after treatment of CGO NF + H_2_O_2_ + NIR.


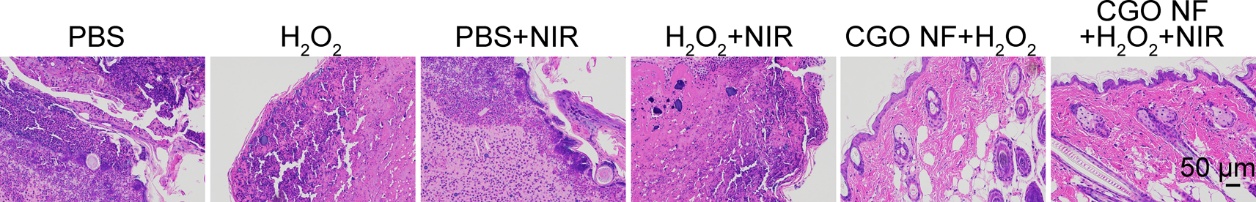


**Figure S46.** H&E staining images of wound bed tissues 10 days after various treatments.


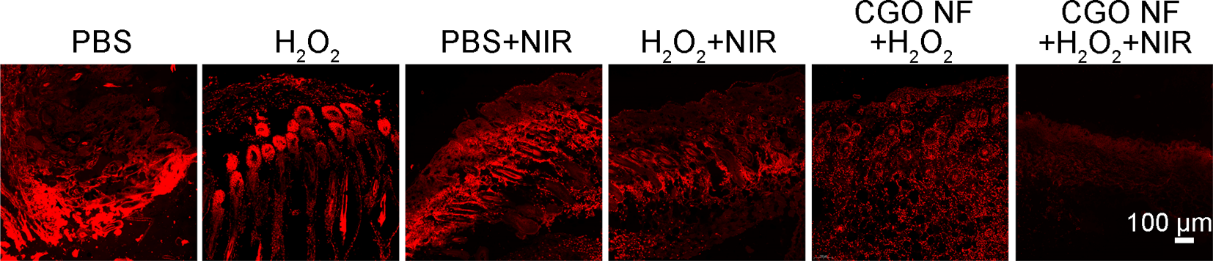


**Figure S47.** Immunofluorescence staining images of DHE in wound bed tissues 5 days following different treatments.

**References**

[1] G. Kresse, D. Joubert, From ultrasoft pseudopotentials to the projector augmented-wave method, Phys. Rev. B 59 (1999) 1758-1775.

[2] J.P. Perdew, K. Burke, M. Ernzerhof, Generalized gradient approximation made simple, Phys. Rev. Lett. 77 (1996) 3865-3868.

[3] M.J. Frisch, G.W. Trucks, H.B. Schlegel, G.E. Scuseria, M.A. Robb, J.R. Cheeseman, G. Scalmani, V. Barone, B. Mennucci, G.A. Petersson, H. Nakatsuji, M. Caricato, X. Li, H.P. Hratchian, A.F. Izmaylov, J. Bloino, G. Zheng, J.L. Sonnenberg, M. Hada, M. Ehara, K. Toyota, R. Fukuda, J. Hasegawa, M. Ishida, T. Nakajima, Y. Honda, O. Kitao, H. Nakai, T. Vreven, J.A. Montgomery, J.E. Peralta, F. Ogliaro, M. Bearpark, J.J. Heyd, E. Brothers, K.N. Kudin, V.N. Staroverov, R. Kobayashi, J. Normand, K. Raghavachari, A.P. Rendell, J.C. Burant, S.S. Iyengar, J. Tomasi, M. Cossi, N. Rega, J.M. Millam, M. Klene, J.E. Knox, J.B. Cross, V. Bakken, C. Adamo, J. Jaramillo, R. Gomperts, R.E. Stratmann, O. Yazyev, A.J. Austin, R. Cammi, C. Pomelli, J.W. Ochterski, R.L. Martin, K. Morokuma, V.G. Zakrzewski, G.A. Voth, P. Salvador, J.J. Dannenberg, S. Dapprich, A.D. Daniels, Ö. Farkas, J.B. Foresman, J.V. Ortiz, J. Cioslowski, D.J. Fox, Gaussian 09, Revision A.02; Gaussian, Inc., Wallingford CT, 2009.

[4] T. Lu, F.W. Chen, Multiwfn: A multifunctional wavefunction analyzer, J. Comput. Chem. 33 (2012) 580-592.

[5] J.W. Liu, H.W. Liang, S.H. Yu, Macroscopic-scale assembled nanowire thin films and their functionalities, Chem. Rev. 112 (2012) 4770-4799.

[6] F.F. Wang, Y. Xing, Z.M. Su, S.Y. Song, Single-crystalline CuGeO_3_ nanorods: synthesis, characterization and properties, Mater. Res. Bull. 48 (2013) 2654-2660.
